# Human visual cortical responses to specular and matte motion flows

**DOI:** 10.3389/fnhum.2015.00579

**Published:** 2015-10-21

**Authors:** Tae-Eui Kam, Damien J. Mannion, Seong-Whan Lee, Katja Doerschner, Daniel J. Kersten

**Affiliations:** ^1^Department of Computer Science and Engineering, Korea UniversitySeoul, South Korea; ^2^Department of Brain and Cognitive Engineering, Korea UniversitySeoul, South Korea; ^3^School of Psychology, UNSW AustraliaSydney, NSW, Australia; ^4^Department of Psychology, University of MinnesotaMinneapolis, MN, USA; ^5^Department of Psychology, Bilkent UniversityAnkara, Turkey; ^6^National Magnetic Resonance Research Center, Bilkent UniversityAnkara, Turkey; ^7^Department of Psychology, Justus-Liebig-University GiessenGiessen, Germany

**Keywords:** visual perception, surface materials, motion flow, functional magnetic resonance imaging (fMRI), classification

## Abstract

Determining the compositional properties of surfaces in the environment is an important visual capacity. One such property is specular reflectance, which encompasses the range from matte to shiny surfaces. Visual estimation of specular reflectance can be informed by characteristic motion profiles; a surface with a specular reflectance that is difficult to determine while static can be confidently disambiguated when set in motion. Here, we used fMRI to trace the sensitivity of human visual cortex to such motion cues, both with and without photometric cues to specular reflectance. Participants viewed rotating blob-like objects that were rendered as images (photometric) or dots (kinematic) with either matte-consistent or shiny-consistent specular reflectance profiles. We were unable to identify any areas in low and mid-level human visual cortex that responded preferentially to surface specular reflectance from motion. However, univariate and multivariate analyses identified several visual areas; V1, V2, V3, V3A/B, and hMT+, capable of differentiating shiny from matte surface flows. These results indicate that the machinery for extracting kinematic cues is present in human visual cortex, but the areas involved in integrating such information with the photometric cues necessary for surface specular reflectance remain unclear.

## 1. Introduction

Experiencing visual qualities, such as the glossiness of polished marble or the smoothness of silk, are an integral part of human conscious experience. The automaticity with which this perceptual process occurs belies the computational difficulty that the brain is faced with in its task to extract meaningful information from the ambiguous retinal signal. The ambiguity lies in whether the pattern of light arriving at the retinae originates from variations in illumination, shape, mesoscale geometrical structure, or the material of the object. Despite this computational challenge humans can effortlessly visually sense dynamic physical properties such as viscosity, elasticity, or stiffness and optical properties such as transparency, glossiness, shininess, or roughness and easily discriminate between material classes. Yet, surprisingly little is understood about how the brain recognizes materials.

While there is a growing body of research on how the visual system extracts optical material qualities such as surface glossiness, roughness or translucency (e.g., Nishida and Shinya, 1998; Adelson, 2001; Dror et al., 2001; Fleming et al., 2004, 2013; te Pas and Pont, 2005; Ho et al., 2006; Motoyoshi et al., 2007; Anderson and Kim, 2009; Doerschner et al., 2010, 2011b; Kim and Anderson, 2010; Olkkonen and Brainard, 2010; Wijntjes and Pont, 2010; Kim et al., 2011, 2012; Marlow et al., 2011, 2012; Zaidi, 2011; Fleming, 2012; Gkioulekas et al., 2013), the majority of this research has focused on visual information available in static images (but see Sakano and Ando, 2008; Wendt et al., 2010). However, image motion can also convey optical material qualities. In a seminal demonstration, Hartung and Kersten (2002) showed that image motion influences how we perceive the material of a rotating object: when surface features are rigidly attached to the object it appears matte, when surface features slide across the shape, consistent with specular flow, the object looks shiny. Critically, when the object was not moving it was ambiguous whether it was shiny or matte (http://gandalf.psych.umn.edu/users/kersten/kersten-lab/demos/MatteOrShiny.html). In subsequent work (Doerschner et al., 2011a), using a combination of image and optic flow analysis, pattern classification and psychophysics, identified three motion cues that the brain could rely on to distinguish between matte and shiny surfaces. Their results revealed a previously unknown use for optic flow in the perception of optical surface material properties. How the brain processes material-dependent image motion, however, is currently unstudied.

In fact, only a few studies have investigated the neural basis of material perception, and these have focused on static image cues to surface material appearance. Electrophysiological studies found neurons in the superior temporal sulcus and anterior inferior temporal cortex to be responsive to surface material properties (e.g., Nishio et al., 2012), whereas neuroimaging studies have identified several loci in low and high level visual areas including V1, V2, V3, V4, posterior inferior temporal cortex, and ventral higher-order visual areas (Peuskens et al., 2004; Cant and Goodale, 2007; Köteles et al., 2008; Cant and Goodale, 2009, 2011; Cavina-Pratesi et al., 2010a,b; Hiramatsu et al., 2011; Okazawa et al., 2012; Wada et al., 2014) with responses in higher level visual areas correlating with perceived similarities of surface material categories, and responses in early visual areas correlating with concurrent changes in simple image features such as spatial frequency or color (Hiramatsu et al., 2011). The first hint that there may be specialized neural mechanisms sensitive to material-specific motion cues came from an experiment by Kam et al. (2012) which found that visual adaptation to a specular rotating object biases subsequently presented objects toward matte appearance.

The study of perceptual or neural responses to optic flow produced by object motion is complicated by the fact that both kinematic and photometric factors contribute to flow. The kinematic deals with the geometric relation between objects and their image projections and is critical for inferring structure. The photometric deals with the relation between the material reflective properties of objects and their images given possibly varying illumination conditions, and is crucial for perceiving material appearance. Reliable estimates of the shape of a rigidly rotating object require the identification of geometric features that are uniquely tied to surface points. Thus, studies of perceived “structure-from-motion” have traditionally relied on kinematic displays composed of moving dots which have a unique relationship to corresponding surface points. However, on inspection such displays convey no information about surface properties such as reflectance or shininess. On the other hand photometric flows are characterized by spatio-temporal changes in intensity which provide information about material properties as well as geometrical structure. While photometric and kinematic factors are not independent (Zang et al., 2010) one can assess the effect of added photometric information.

Here, our aim was to investigate the brain processing underlying the perception of specular reflectance from motion in human observers. We used functional magnetic resonance imaging (fMRI) to infer the magnitude of brain activation across posterior visual cortex to rotating blob-like objects. Following Doerschner et al. (2011a), we rendered the objects in image sequences that yield a perceptual impression of a matte or shiny specular reflectance when set in motion. We also identified the flow structure of such sequences and used dots to create presentation conditions that mimic the matte and specular flows but lack the perception of surface specular reflectance. Our key prediction was that areas of human visual cortex that are sensitive to specular reflectance from motion would show an interaction between the rendering type (image or dot) and the flow type (matte or shiny).

## 2. Materials and methods

### 2.1. Participants

Nine participants, each with normal vision, participated in the current study. Each participant gave their informed written consent and the study conformed to safety guidelines for MRI research and was approved by the Institutional Review Board at Korea University. One participant was excluded from analysis due to difficulties in defining his/her retinotopic visual areas, and the analysis presented here is derived from the remaining eight participants.

### 2.2. Apparatus

Functional imaging was conducted using a Siemens 3T-Trio magnet (Erlangen, Germany) with a 32-channel head coil. To allow participants to have unrestricted viewing of the display through each eye, the coil was operated with the lower 20 elements only. Images were collected with a T^*^_2_ sensitive gradient echo imaging pulse sequence (TR = 3 s, *TE* = 30 ms, *delay in TR* = 0.8 s, flip angle = 90°, *matrix* = 96 × 96, GRAPPA acceleration factor = 2, FOV = 192 × 192 mm, partial Fourier$=\frac{7}{8}$, *voxel size* = 2 mm isotropic) in 35 interleaved oblique coronal slices covering the occipital lobes. Stimuli were displayed on an LCD monitor (“BOLDscreen,” Cambridge Research Systems, Kent, UK) with a spatial resolution of 1920 × 1200 pixels, temporal resolution of 60 Hz, and mean luminance of 450 cd/m^2^. The monitor output was linearized via correction of luminance values measured with a ColorCAL MKII colorimeter (Cambridge Research Systems, Kent, UK). The screen was viewed through a mirror mounted on the head coil at a distance of 112 cm, giving a viewing angle of 26.0° × 16.4°. Stimuli were displayed using PsychToolbox (Brainard, 1997; Pelli, 1997; Kleiner et al., 2007) on a Macbook Pro driving an Intel HD Graphics 4000 video card. As detailed below, analyses were performed using FreeSurfer 5.1.0 (Dale et al., 1999; Fischl et al., 1999), FSL 4.1.6 (Smith et al., 2004), and AFNI/SUMA (2013/05/22; Cox, 1996; Saad et al., 2004).

### 2.3. Stimuli

Four stimulus conditions, each based on a single blob-like object, were developed: a *rendering* class with two conditions corresponding to photometric and kinematic displays, which we call “image” and “dot” flows, respectively. These conditions were crossed with a *material flow* class with two conditions referred to as “matte” and “shiny.”

**Figure 1:**
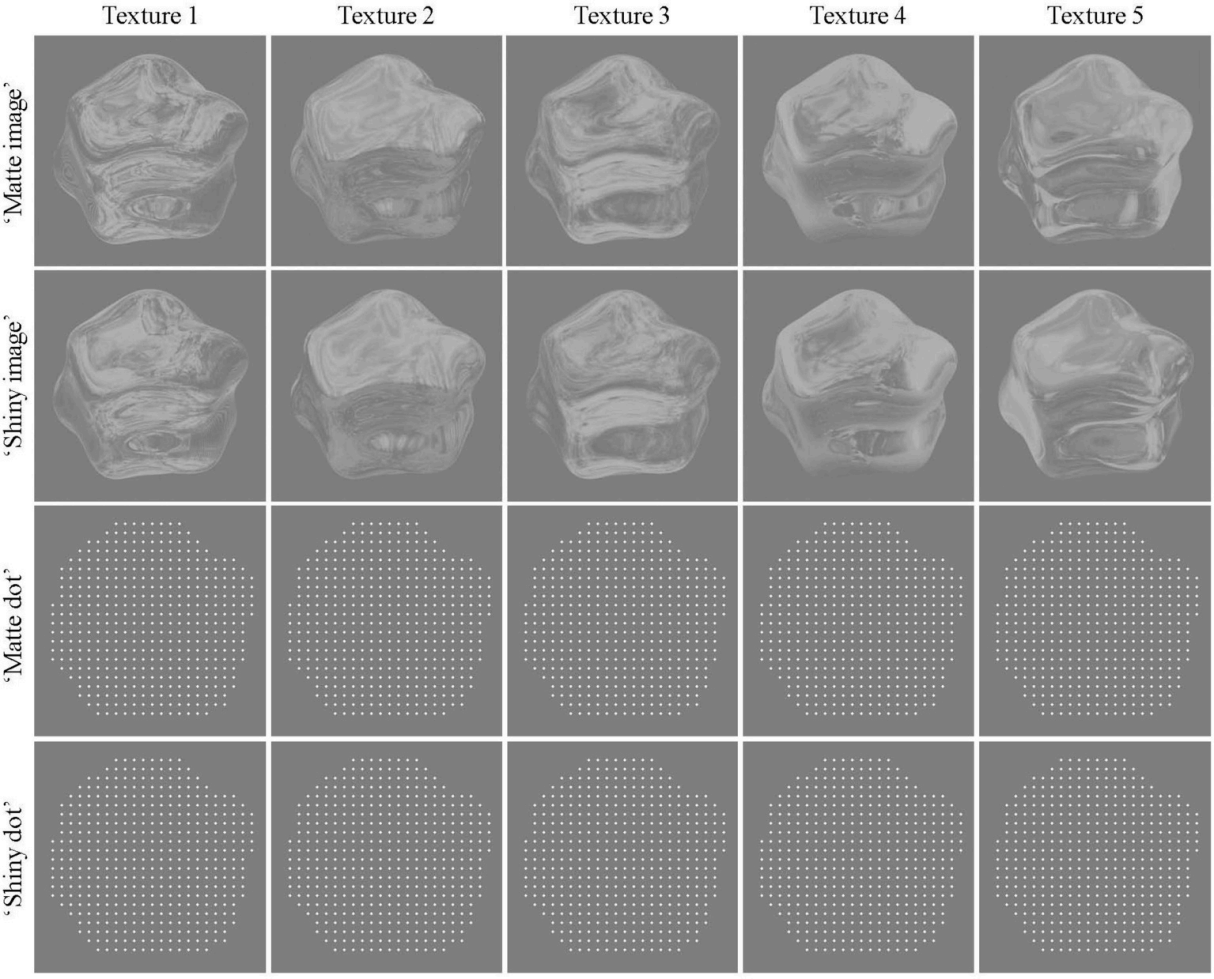
**Example frames for “matte” and “shiny” rendered images and dots**. Columns show the set of environment maps that were applied to the object, and rows show the texture applied as diffuse (“matte”) and specular (“shiny”) reflectance with each rendering method. In these static images, the “shiny” and “matte” conditions are difficult to discriminate. In sequences of rotation, however, they are readily perceived as having different reflectance properties (see Supplementary Movies 1, 2).

“Image” renderings were created with Radiance 3D (Larson et al., 1998) using the environment-mapping techniques described in Debevec (2002). Objects were either rendered as specularily-reflecting or as textured and diffusely reflecting. For the latter, the specular reflection from one particular view point was “stuck on” to the object's surface creating a matte, textured appearance when the object was rotating. See Doerschner et al., 2011a for details of the object generation and rendering procedure, and see Figure 1 for stimulus examples.

Five environment maps were generated by combining several maps of the Debevec (2002) database using an array of techniques in Adobe Photoshop such as blending, duplicating, and reflecting over the horizontal axis. This was done in order to maximize spatial complexity in the resulting environment map, and to obtain optimal optic flow estimates. The object was assigned a material property of either diffuse or specular reflectance. Such material properties are difficult to distinguish when viewed statically, as evident in Figure 1 and quantified in Supplementary Figure 1, and this ambiguity was further enhanced by histogram equalization and contrast reduction. The luminance histogram was equalized using the method proposed by Gonzalez and Woods (2008). This approach essentially reduces the skewness in the luminance histogram, and thus removes a (static) image cue that has been shown to correlate with a glossy appearance (Motoyoshi et al., 2007). As a next step, we decreased the intensity of pixels above a value of 128 using the following procedure: we computed the difference that a given pixel intensity has with respect to 128, and multiplied this difference by 0.4. The new pixel value was then obtained by adding the attenuated difference to 128, and rounding the result. As an example a pixel value of 255 would be reduced to 179 (128 + (255 − 128) × 0.4). This manipulation reduced image contrast, by attenuating the intensity of particular bright regions on the object (such as specular highlights), thus further reducing the effects of static cues to perceived glossiness (Marlow et al., 2011). By these two processing steps the mean luminance and contrast of all stimuli (“shiny” and “matte”) was effectively equated to a value of 141 and 0.2, respectively.

We define photometric cues on Section 1 as those, signaling the relation between material reflective properties of objects and their images given varying illumination conditions. Luminance histogram skewness and image contrast are only two potential correlates of shiny appearance (see Chadwick and Kentridge, 2015), thus while we might have reduced photometric cues to glossiness we did not eliminate all. In particular the idiosyncratic spatial structure (e.g., compression at high curvature points) in the images of specular objects remained intact (e.g., Fleming et al., 2004).

More importantly, we wanted to create a baseline stimulus where the surface material is ambiguous in the static case [as in the Hartung and Kersten (2002) demo, i.e., both interpretations, “shiny” and “matte,” are equally likely; also see Supplementary Figure 1]. Had our objects already looked shiny in the static case, we would have introduced an asymmetry for the moving stimuli: the motion of “sticky” reflections patterns (matte appearance) would have been surprising and in conflict with the photometric cues (say high positive skewness), whereas the specular motion would not have this conflicting information. In order to avoid this confounding asymmetry we performed the above described image manipulations.

The kinematic dot renders were created by applying a phase-based optical flow method (Gautama and Van Hulle, 2002) to the image renders. Dots (white diamonds, 0.1° visual angle in diameter) were initially placed at uniform locations in the two-dimensional area occupied by the object (9 dots/° visual angle^2^ density), and each dot was then moved on each frame by an estimate of its underlying flow field (see Supplementary Movies 3, 4). Due to the lack of photometric information, the shiny/dot and matte/dot conditions are not perceived as shiny or matte. Both image and dot renders do, however, retain the differences between shiny and matte stimuli in coverage, divergence, and shape reliability cues that were reported by Doerschner et al. (2011a); see Supplementary Figures 2–4 and Supplementary Tables 1–3. The details of the methods for calculating the features are described in Doerschner et al. (2011a).

### 2.4. Experimental procedures

**Figure 2:**
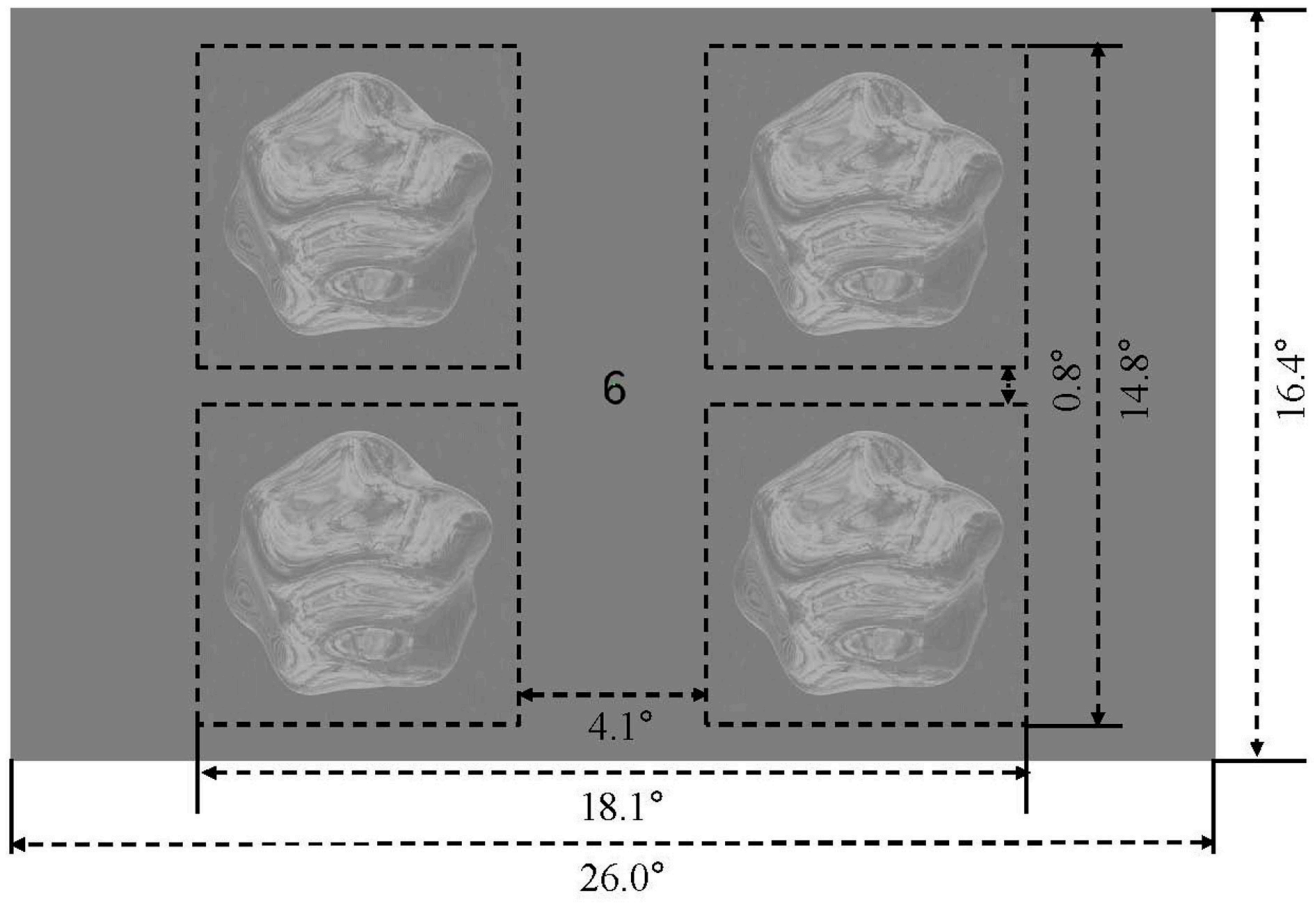
**Display and stimulus layout**. Four rotating objects were presented in the visual field quadrants, surrounding a central fixation marker.

The four stimulus conditions (matte/shiny image flows, matte/shiny dot flows) were presented in a block design. Within each stimulus block, four objects, each rendered with the the same environment map (assigned from a set of five maps in pseudorandom order across blocks) and being approximately 7.0° in diameter, were centered at 6.9° eccentricity within the visual field quadrants (see Figure 2). Each object rotated back and forth eight times over the course of a 15 s block, with each rotation traveling 15°. The object in a given visual field quadrant rotated about the same axis over the course of the experiment, and the three cardinal axes and one oblique axis were assigned to the four visual field quadrants (see Supplementary Movie 5). Blocks were ordered in sequences in which the four stimulus condition blocks were followed by a blank block, with the arrangement of stimulus blocks chosen such that each condition was preceded an equal number of times by each of the other conditions. There were four such sequences per run, and an additional blank block was appended to the run order, giving a run duration of 315 s (105 volumes). Each participant completed 12 runs, collected within a single session.

A demanding foveal task was used to control fixation, and divert attention from the appearance of the objects. Throughout each run, a sequence of digits, which were randomly chosen from zero to nine and of random polarity (black or white), was presented at center of the screen. Participants were instructed to press a button when one of two target digits appeared in the sequence. The foveal targets, which had opposite polarities and differing digits, were introduced to the participants at the beginning of each run.

### 2.5. Anatomical acquisition and processing

A T_1_-weighted anatomical image (sagittal MP-RAGE, 1mm isotropic resolution) was collected from each participant in a separate session. FreeSurfer (Dale et al., 1999; Fischl et al., 1999) was used for segmentation, cortical surface reconstruction, and surface inflation and flattening of each participant's anatomical image.

### 2.6. Visual area definition

**Figure 3:**
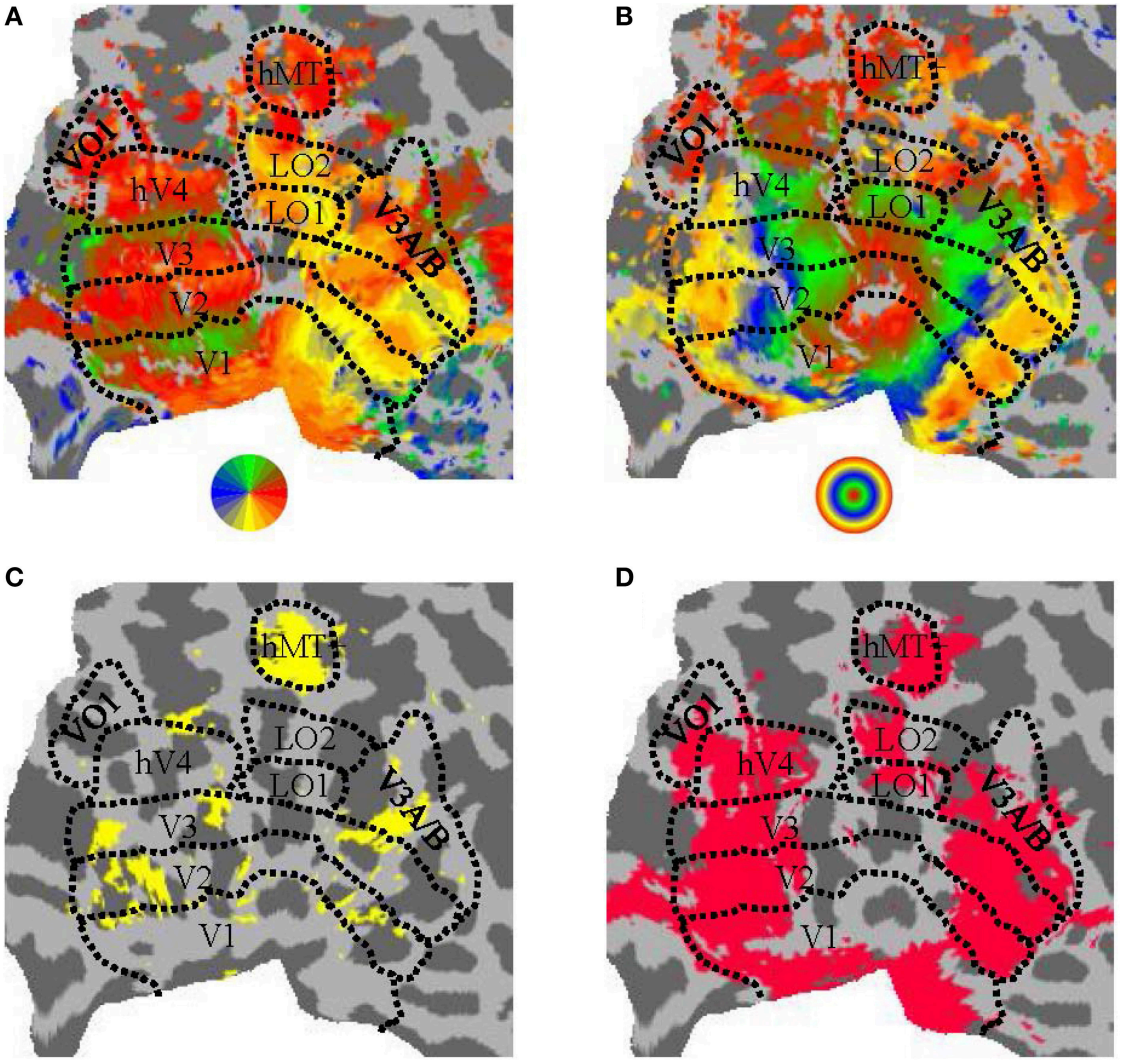
**Parcellation of human visual cortex of an example participant**. **(A)** Angular visual field preference obtained from rotating wedge stimulation, with a threshold of 10dB BOLD signal-to-noise-ratio (where “signal” was defined as the power of the BOLD timeseries at the wedge rotation frequency and “noise” was defined as the average power in the two neighboring frequency bins). **(B)** Eccentricity visual field preference obtained from expanding/contracting ring stimulation, with a threshold as in **(A)**. **(C)** Regions significantly (*p*_*uncorrected*_ < 0.01) responsive to a moving (expanding/contracting) > static dots stimulus. **(D)** Regions significantly responsive to all conditions against blank condition contrast (*p*_FDR_ < 0.001). All panels show a flattened representation of an example participant's posterior left hemisphere. Colors in **(A,B)** correspond to the visual field legend shown below the panel.

In a separate session, standard protocols (Sereno et al., 1995; DeYoe et al., 1996; Engel et al., 1997; Larsson and Heeger, 2006; Hansen et al., 2007; Schira et al., 2007; Bressler and Silver, 2010) were used for defining retinotopic visual areas of the brain. Participants observed a clockwise/anti-clockwise rotating wedge stimulus during four runs and expanding/contracting ring stimulus during two runs. We manually defined the visual areas V1, V2, and V3 (Dougherty et al., 2003), hV4 (Wade et al., 2002; Goddard et al., 2011), LO1/2 (Larsson and Heeger, 2006), and VO1 (Brewer et al., 2005) based on the visual field preferences established from phase-encoded analysis (see Figure 3).

We also functionally defined the human MT complex (hMT+; Tootell et al., 1995) and Lateral Occipital Complex (LOC; Malach et al., 1995) via two runs of moving (expanding/contracting) and static low-contrast dots and two runs of a Face-Place-Object (FPO) paradigm (Cant and Goodale, 2011), respectively. For the FPO paradigm, we collected face and object images from the Carnegie Mellon University database (Harvey and Burgund, 2012) and place images from the Caltech database (Andrews et al., 2010).

The relevant localizer contrast from a GLM analysis (motion > static for hMT+, objects > faces and places for LOC) was visualized on the flattened cortical surface of each participant and hemisphere. The threshold on the contrast statistic was manually adjusted to estimate the extent of hMT+ and LOC, with the location of hMT+ and LOC identified as the cluster that matched the expected anatomical location and the expected location with respect to the retinotopically-defined visual areas. The ROIs were then drawn manually as a closed and filled region on the cortical surface (see Figure 3C for an example ROI definition for hMT+).

### 2.7. Pre-processing

Functional images were motion corrected using AFNI, with reference to the volume acquired closest in time to a within-session fieldmap image, and resampled with heptic interpolation before being unwarped using FSL to correct geometric distortions introduced by magnetic field inhomogeneities. No slice-timing correction was applied. The participant's anatomical image was then coregistered with a mean of all functional images via AFNI's align_epi_anat.py, using a local Pearson correlation cost function (Saad et al., 2009) and six free parameters (three translation, three rotation). Coarse registration parameters were determined manually and passed to the registration routine to provide initial estimates and to constrain the range of reasonable transformation parameter values. The motion-corrected and unwarped functional data were then projected onto the cortical surface by averaging between the white matter and pial boundaries (identified with FreeSurfer) using AFNI/SUMA. No specific spatial smoothing was applied. All analysis was performed on the nodes of this surface domain representation in the participant's native brain space.

### 2.8. Univariate analysis

Stimulus blocks for each condition were modeled as boxcars and convolved with SPM's canonical haemodynamic response function. Legendre polynomials up to the third degree were included as additional regressors, for each run, to detrend for low-frequency noise. The GLM was estimated via AFNI's 3dREMLfit, which accounts for noise temporal correlations via a voxelwise ARMA(1,1) model.

The stimulus condition beta weights obtained from the GLM were converted to Percent Signal Change (PSC) via division by the average of the synthesized baseline timecourse (derived from the Legendre polynomial regressors). For each visual area, such percent signal change values were then averaged across the cortical surface nodes within the area that showed above-baseline responses to visual stimulation (identified by an all stimulus >0 contrast, *p*_FDR_ < 0.001).

The mean PSC value across participants within each area were normalized by subtracting each participant's mean response across stimulus conditions and adding the grand mean across participants and conditions (Cousineau, 2005). A Two-way within-subjects ANOVA was then conducted for each visual area, with flow (matte, shiny) and rendering (image, dots) as fixed factors and participants as a random factor.

### 2.9. Multivariate pattern analysis (MVPA)

The timeseries for each participant and run were first high-pass filtered with Legendre polynomials up to the third degree. An amplitude was then estimated for each block as the mean signal within its five volumes (15 s), shifted by two volumes (6 s) to compensate for the delayed hemodynamic response. The amplitude estimates within each run were then normalized (*z*-scored). This procedure produced 192 responses per participant for each node on the cortical surface; four responses for each of four conditions in each of 12 runs.

The MVPA was performed separately for each participant and visual area, and was implemented using a 12-fold leave-one-run-out strategy in which the responses from a given run were designated (in turn) to form the “test” set and the remaining runs to form the “training” set. In each fold, separate linear support vector machines (SVMs) were trained for image and dot renderings on labeled examples of matte and shiny flow response patterns. Each training set thus consisted of 88 examples, with matte and shiny flow examples equally represented. Flow discrimination accuracy was then estimated by using the trained SVMs to predict the flow condition of test set examples from the same (within-class) or different (between-class) rendering. SVMs were implemented with libsvm 3.17 (Chang and Lin, 2011) via Matlab 8.1.0.604 (The Mathworks Inc., Natick, MA). The accuracy was based on the proportion of hits and false alarms after aggregation of the 12-folds, and expressed in *d*′ units. The *d*′ calculation included an addition of 0.5 to all hit and false alarm counts and an addition of 1 to the number of trials in each condition class, in order to accommodate extreme hit or false alarm rates (Stanislaw and Todorov, 1999).

Features were selected for inclusion in the response pattern for a given visual area based on the *t*-value of the all stimulus > blank screen contrast performed in the univariate analysis. The surface nodes within a given visual area were ranked in descending order based on the magnitude of this localizing *t*-value, and the MVPA procedure was performed with patterns formed from including increasing numbers of such ranked nodes (from *n* = 10 to *N*, where *N* is the number of nodes with statistically significant *t*-values at *p* < 0.05, one-tailed, uncorrected) in 10 node increments. The variation in MVPA performance with increasing nodes was summarized via a least-squares fit to the function:

(1) $$\begin{array}{l} p=a\left(1-e^{\frac{-n}{c}}\right) \end{array}$$

where *p* is the performance level (*d*′), *n* is the number of included nodes, and *a* and *c* are fitted parameters that describe the asymptotic performance (*a*) and curvature (*c*). The classification accuracy for a given participant, visual area, training class (image, dot), and testing class (within-class, between-class) was then taken as either the fitted asymptote or, in the case of unsuccessful fit, the mean accuracy over nodes. The classification accuracy and fitted performance levels with increasing nodes are shown in Supplementary Figures 5–13.

### 2.10. Multiple comparisons correction

The largely exploratory nature of the research question investigated in this study motivates the testing of hypotheses in multiple candidate regions in the posterior vision-sensitive area of the brain. This approach necessitates consideration of the inflationary effect of performing statistical analyses in multiple regions-of-interest (visual areas) on the rate of false-positive inference. Here, we adopted a false discovery rate (FDR; Benjamini and Hochberg, 1995) strategy to help reduce this multiple comparisons problem. Specifically, we adjusted the probability outcomes of each statistical test conducted across multiple visual areas via the FDR procedure, and the resulting *p*_FDR_ values were then evaluated at a criterion of 0.05 for statistical significance (with the exception of the localizer contrast, which was evaluated at 0.001).

## 3. Results

We examined the response characteristics of the low and mid-level regions of human visual cortex during observation of blob-like objects with motion properties consistent with different surface attributes (shiny and matte). The objects were rendered either as dynamic intensity flows (the photometric or “image” condition) or as moving light points (the kinematic or “dot” condition). Photometric presentation supports the perception of matte or shiny surface attributes, whereas kinematic presentation, despite containing similar flow patterns, does not evoke a perception of matte or shiny surface structure. The renderings were presented in four quadrants of the visual field (see Figure 2) while fMRI was used to measure the BOLD activity from within the posterior region of human visual cortex. This stimulus presentation evoked significant activity levels (*p*_FDR_ < 0.001), aggregated over shiny and matte renderings and dot flows and compared with a blank screen baseline, within low-level visual areas V1, V2, and V3, dorsal areas LO1, LO2, V3A/B, and hMT+, and ventral areas hV4 and VO1. We also evaluated the anterior subdivision of the LOC, defined functionally as preferring images of objects over images of faces and houses, but it was not activated consistently across participants and was therefore not analyzed further.

### 3.1. Response amplitude

**Figure 4:**
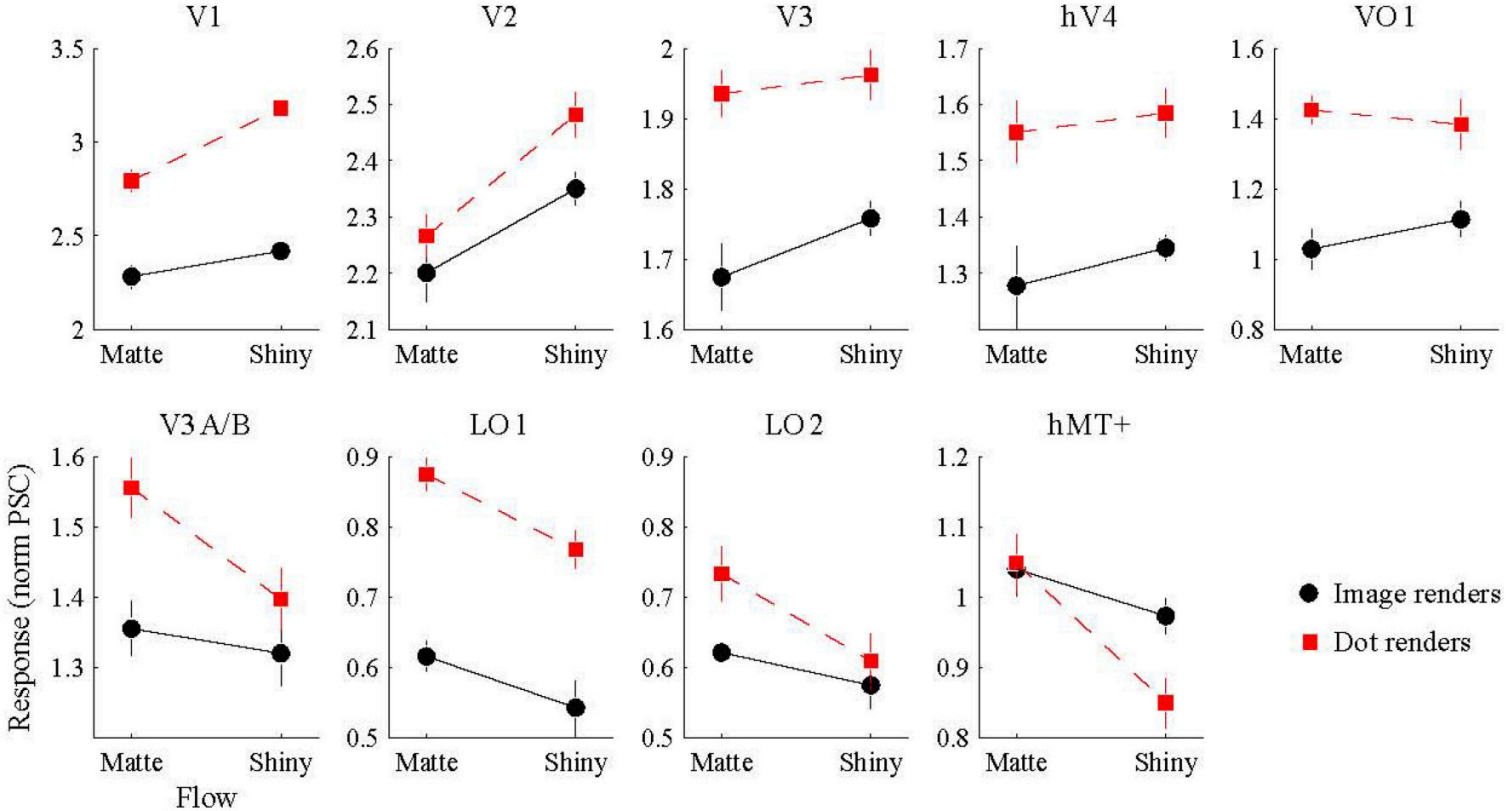
**BOLD signal amplitude evoked by image and dot renders of shiny and matte flows**. Each panel shows the magnitude of BOLD response (normalized percent signal change), averaged over participants (error bars indicate SEM).

We conducted a Two-way ANOVA (flow class: matte, shiny; rendering: image, dot) on the mean response amplitude elicited in each visual area. We predicted that the perceptual sensitivity to material properties from motion would be related to the activity levels in the low and mid-level regions of human visual cortex, and that this would be demonstrated via an interaction between the motion flow class (matte, shiny) and the rendering type (image, dot). However, as shown in Figure 4, such an interaction was not evident in any of the investigated visual areas (all *p*_FDR_ > > 0.05; see Table 1, for details).

**Table 1:** **Results of Two-way ANOVA for each visual area, conducted on the average signal amplitude for each participant**.

**Area**	**Flow (shiny, matte)**			**Rendering (image, dots)**			**Flow** × **Rendering**		
	***F*_(1, 7)_**	***p***	***p*_FDR_**	***F*_(1, 7)_**	***p***	***p*_FDR_**	***F*_(1, 7)_**	***p***	***p*_FDR_**
V1	37.62	< 0.001	0.004	89.87	< 0.001	< 0.001	3.85	0.090	0.509
V2	20.22	0.003	0.013	3.03	0.126	0.161	0.43	0.531	0.710
V3	4.49	0.072	0.092	17.72	0.004	0.012	0.39	0.552	0.710
hV4	1.54	0.254	0.286	8.69	0.021	0.039	0.11	0.747	0.747
VO1	0.31	0.593	0.593	10.43	0.014	0.033	2.28	0.175	0.509
V3A/B	4.81	0.064	0.092	4.58	0.070	0.105	1.76	0.226	0.509
LO1	9.72	0.017	0.038	43.42	< 0.001	0.001	0.21	0.659	0.741
LO2	6.33	0.040	0.072	2.69	0.145	0.163	0.84	0.389	0.701
hMT+	13.41	0.008	0.024	1.42	0.272	0.272	1.98	0.202	0.509

We then investigated the potential main effects of flow (matte, shiny) and rendering (image, dot). There was a significant main effect of flow class for the mean BOLD response amplitudes in V1 [*F*_(1, 7)_ = 37.62, *p*_FDR_ = 0.004], V2 [*F*_(1, 7)_ = 20.22, *p*_FDR_ = 0.013], LO1 [*F*_(1, 7)_ = 9.72, *p*_FDR_ = 0.038], and hMT+ [*F*_(1, 7)_ = 13.41, *p*_FDR_ = 0.024]. Responses were greater for shiny than matte flows in V1 (difference mean = 0.26, SEM = 0.04) and V2 (difference mean = 0.18, SEM = 0.04) while response were greater for matte than shiny in LO1 (difference mean = 0.09, SEM = 0.03) and hMT+ (difference mean = 0.13, SEM = 0.04). The main effect of flow class was not significant in the other visual areas under investigation (*p*_FDR_ > 0.05; see Table 1).

There was also a significant main effect of rendering class for the mean BOLD response amplitudes in V1 [*F*_(1, 7)_ = 89.87, *p*_FDR_ < 0.001], V3 [*F*_(1, 7)_ = 17.72, *p*_FDR_ = 0.012], hV4 [*F*_(1, 7)_ = 8.69, *p*_FDR_ = 0.039], VO1 [*F*_(1, 7)_ = 10.43, *p*_FDR_ = 0.033], and LO1 [*F*_(1, 7)_ = 43.42, *p*_FDR_ = 0.001]. Responses were greater for dots than images in each of these areas (V1 difference mean = 0.64, SEM = 0.07; V3 difference mean = 0.23, SEM = 0.06; hV4 difference mean = 0.26, SEM = 0.09; VO1 difference mean = 0.33, SEM = 0.10; LO1 difference mean = 0.24, SEM = 0.04). The main effect of rendering class was not significant in the other visual areas under investigation (*p*_FDR_ > 0.05; see Table 1).

### 3.2. Response pattern

**Figure 5:**
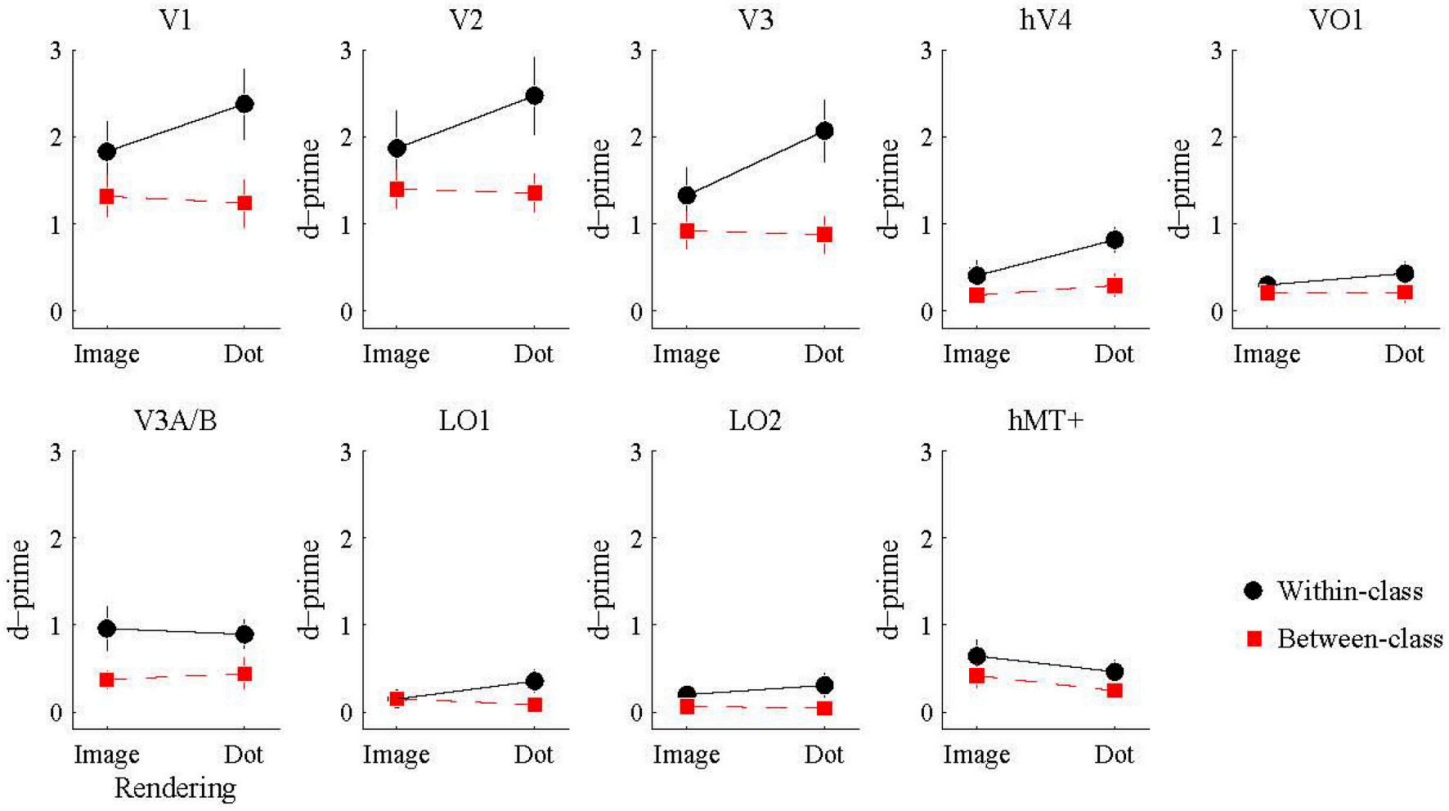
**Accuracy of multivariate pattern classification of flow type (shiny/matte) in each visual area**. Within-class (black) accuracy denotes the level at which the pattern of responses to a given rendering class (image/dot) can distinguish the material flow type (shiny/matte) of the same rendering class. Between-class (red) accuracy denotes the level at which the pattern of responses to a given rendering class (image/dot) can distinguish the material class (shiny/matte) of the other rendering class. Points and lines show mean and SEM over participants, respectively, in *d*′ units.

We also investigated whether the multivariate pattern of responses within each visual area could discriminate between shiny and matte flows. We employed a pattern classification procedure to test for visual areas with a representation of surface material from motion flows. For each visual area, a classifier was trained with activation patterns from a particular rendering class (image or dot) and then tested with either the same or different rendering class (within or between-class classification). The rationale was that a visual area representing surface material would be significantly better at discriminating shiny and matte flows for within-class classifications with image renderings, since that is the only combination in which surface material perception differs between shiny and matte in the same way between training and testing. However, as shown in Figure 5, this interaction between rendering class (image, dot) and classification type (within, between) was not statistically significant in any of the visual areas under consideration (all *p*_FDR_ > 0.05; see Table 2, for details).

**Table 2:** **Results of Two-way ANOVA for each visual area, conducted on the accuracy (***d***′) for each participant**.

**Area**	**Classification (within, between)**			**Rendering (image, dots)**			**Classification** × **Rendering**		
	***F*_(1, 7)_**	***p***	***p*_FDR_**	***F*_(1, 7)_**	***p***	***p*_FDR_**	***F*_(1, 7)_**	***p***	***p*_FDR_**
V1	25.26	0.002	0.011	3.02	0.126	0.313	10.32	0.015	0.133
V2	11.49	0.012	0.021	2.72	0.143	0.313	4.91	0.062	0.187
V3	21.16	0.002	0.011	4.61	0.069	0.310	6.27	0.041	0.184
hV4	18.10	0.004	0.011	10.62	0.014	0.125	2.60	0.151	0.226
VO1	3.52	0.103	0.132	0.97	0.358	0.537	2.96	0.129	0.226
V3A/B	14.28	0.007	0.016	0.00	0.998	0.998	0.26	0.627	0.705
LO1	1.13	0.323	0.323	0.64	0.451	0.580	2.90	0.132	0.226
LO2	7.08	0.032	0.049	0.42	0.538	0.605	0.42	0.539	0.693
hMT+	2.31	0.173	0.194	2.29	0.174	0.313	0.00	0.980	0.980

We then considered the main effects of rendering class (image, dot) and classification type (within, between) on the observed classification accuracies. None of the visual areas under consideration had a significant main effect of rendering class (all *p*_FDR_ > 0.05). The main effect of classification type was significant in V1 [*F*_(1, 7)_ = 25.26, *p*_FDR_ = 0.011], V2 [*F*_(1, 7)_ = 11.49, *p*_FDR_ = 0.021], V3 [*F*_(1, 7)_ = 21.16, *p*_FDR_ = 0.011], hV4 [*F*_(1, 7)_ = 18.10, *p*_FDR_ = 0.011], V3A/B [*F*_(1, 7)_ = 14.28, *p*_FDR_ = 0.016], and LO2 [*F*_(1, 7)_ = 7.08, *p*_FDR_ = 0.049]. As shown in Figure 5, accuracies were significantly greater for within-class than between-class classification in these areas.

Finally, we examined the ability of each visual area and classification condition to discriminate shiny and matte flows at levels significantly greater than chance. All of the visual areas under consideration were able to perform within-class classification (image → image, dot → dot) significantly greater than chance (all *p*_FDR_ < 0.05 except LO2 where *p*_FDR_ = 0.057; see Table 3, for details). Visual areas V1, V2, V3, V3A/B, and hMT+ were also able to perform between-class classification (image → dot, dot → image) significantly greater than chance (all *p*_FDR_ < 0.05). The between-class classification performance was less reliable for hV4 and VO1 (*p*_FDR_ between 0.051 and 0.067), and not significantly greater than chance in LO1 and LO2 (all *p*_FDR_ > 0.05).

**Table 3:** **Results of a one-sample ***t***-test against a chance level of ***d***′ accuracy of 0 for each visual area, conducted on the accuracy of multivariate pattern classification**.

	**Image**→**Image**			**Dot**→**Dot**			**Image**→**Dot**			**Dot**→**Image**		
**Area**	*t*_7_	*p*	*p*_FDR_	*t*_7_	*p*	*p*_FDR_	*t*_7_	*p*	*p*_FDR_	*t*_7_	*p*	*p*_FDR_
V1	4.94	0.001	0.008	5.55	< 0.001	0.001	5.28	0.001	0.003	4.23	0.002	0.006
V2	4.05	0.002	0.009	5.19	0.001	0.001	5.86	< 0.001	0.003	5.78	< 0.001	0.003
V3	3.89	0.003	0.009	5.40	0.001	0.001	4.10	0.002	0.007	3.87	0.003	0.007
hV4	2.32	0.027	0.030	5.32	0.001	0.001	2.06	0.039	0.054	2.16	0.034	0.051
VO1	2.32	0.027	0.030	2.90	0.011	0.015	2.01	0.042	0.054	1.87	0.052	0.067
V3A/B	3.57	0.005	0.010	5.08	0.001	0.001	3.32	0.006	0.014	2.32	0.027	0.048
LO1	2.63	0.017	0.025	2.66	0.016	0.018	1.50	0.088	0.099	1.39	0.104	0.117
LO2	1.81	0.057	0.057	2.12	0.036	0.036	1.07	0.160	0.160	1.07	0.161	0.161
hMT+	3.22	0.007	0.013	3.45	0.005	0.008	2.75	0.014	0.026	5.16	0.001	0.003

## 4. Discussion

We measured the responses of visual areas to rotating 3D objects, with photometric or kinematic flows corresponding to shiny or matte objects, while observers were engaged in a demanding fixation task. With almost all of the previous work investigating the cortical processing of material properties with static images (Cant and Goodale, 2007, 2009; Hiramatsu et al., 2011), we focused here on dynamic information for surface reflectance (Doerschner et al., 2011a). While we find no obvious candidates in human low and mid-level visual cortex for an explicit representation of specular reflectance from motion, we do find several areas with response properties that are modulated by the structure of matte and shiny flows.

### 4.1. Representation of specular reflectance from motion

We did not observe an interaction between rendering type (image, dot) and flow (matte, shiny) in the mean response amplitudes of any of the visual areas under consideration. We also did not observe a difference in the accuracy of pattern classification performance that would be indicative of a representation of specular reflectance from motion in any of the visual areas.

However, the results outlined above do not necessarily indicate that the visual areas investigated in the current study are unable to represent the perception of specular reflectance from motion. Such a representation may produce differences in response amplitude that are too small to be recovered with the current design, or may be present at a spatial scale that is not visible to the resolution of our fMRI measurements. Furthermore, it is possible that the extraction of specular reflectance from motion requires observer attention to be directed at the stimulus. Our wariness of a attentional confound, in which shiny surfaces are potentially more engaging than matte surfaces, led us to direct our observers' attention to an unrelated task at fixation. However, a potential consequence of this attentional focus is a lack of activation of cortical pathways that are involved in extracting the surface properties from the motion flows. This could perhaps be assessed behaviorally in future studies using an adaptation paradigm (Kam et al., 2012) with differing attentional demands.

### 4.2. Correlates of specular reflectance motion

We did find several areas in low and mid-level human visual cortex that were affected by whether the flow was consistent with a shiny or matte surface. Shiny and matte flows differ on many dimensions, which raises the question of which aspects of the motion signals are affecting the response properties of the different visual areas. For example, shiny and matte flows can differ in the statistical distribution of velocity parameters (mean and variance of speed and direction; cf. Doerschner et al., 2011b), luminance contrast, and higher-order regularities. We next consider the potential contribution of such dimensions to the visual regions under investigation.

#### 4.2.1. Effect of matte vs. shiny flow class on average responses

While areas V1, V2, LO1, and hMT+ all had significantly different levels of mean activity for shiny and matte flows, V1 and V2 showed larger responses to shiny than to matte flows, whereas LO1 and hMT+ showed the opposite pattern (Figure 4).

If shiny and matte objects contain different levels of motion energy, that could potentially account for the differences in activity levels we observed in early visual areas. We checked this possibility by computing motion energy for all of our stimuli using Gabor filters (Adelson and Bergen, 1985). While the motion energy between shiny and matte photometric flows did not differ significantly, we found that motion energy for matte dot flows were significantly larger than for shiny dot flows (Supplementary Figure 14 and Supplementary Tables 4, 5). If cortical areas were sensitive solely to motion energy we would expect a significant interaction term for rendering type and material class for identified cortical areas. This was not, however, what we found. Thus, while motion energy might explain the general larger responses of cortical areas to dot flows^1^, they cannot account for the overall observed response pattern.

Macaque V1 (Priebe et al., 2006) and MT (Priebe et al., 2003) contains neurons selective for stimulus speed, and a difference in speed distributions between shiny and matte stimuli could potentially introduce differences in response magnitude into the observed fMRI responses. We computed speed histograms to evaluate such a possibility and find no difference in mean speed between shiny and matte flows (Supplementary Figure 15), making it unlikely that speed is responsible for the effects observed in low-level areas.

Inspecting the corresponding motion direction histograms in Supplementary Figure 15 it becomes apparent that the distributions for matte flows form a cluster around a dominant direction of motion whereas shiny flows tend to be distributed more uniformly—owing to the large variations of flow direction in specular flow (Doerschner et al., 2011a). Considering the different receptive field sizes in V1/V2 compared to hMT+/LO1 (Mikami et al., 1986; Amano et al., 2009) we might be able to partially explain the opposing response patterns to matte and shiny flows in these areas: due to their small receptive field sizes, non-overlapping neural populations in V1/V2 might respond their preferred motion, creating large net response. In hMT+, however, due to the larger receptive field sizes, and opponent processes (Heeger et al., 1999) a given neural population may have preferred and non-preferred direction in their receptive field present, thus creating an overall decreased response. By the same argument we would expect decreased net response in V1/V2 to matte flows and a relatively increased net response in hMT+/LO1. Though variability in motion direction can be related to motion coherency these two are not the same concept^2^. Thus, we consider the latter as a potential explanation of our results next.

Shiny and matte flows, both kinematic and photometric, differ with respect to the degree of motion coherency. There is large literature which has studied behavioral and neural responses to structure-from-motion stimuli composed of dots whose coherency is manipulated by assigning random velocities to varying proportions of the dots. While this dimension does not map simply on to matte vs. shiny flows, coherency is correlated with distortions in the appearance of specular flows (Doerschner et al., 2011a). Specifically Doerschner et al. (2011a) showed that distortion manifests itself in the degree of expansion/contraction of optic flow (“divergence”), the degree to which image features could be unambiguously tracked across time (“trackability”), and the degree to which features were consistent with rigid-body rotation (“shape reliability”), for matte and shiny surfaces. Both decreased trackability and shape reliability (and perhaps to a lesser extent divergence) are also characteristic of decreased coherence in random dot displays. It has been shown that V1 shows greater activation for random dot motion than for translational coherent motion (Braddick et al., 2001, but see Morrone et al., 2000), wheras areas V5/hMT+ responses increase with increases in motion coherence (Rees et al., 2000; Braddick et al., 2001; Händel et al., 2007 but also see McKeefry et al., 1997; Paradis et al., 2000; Smith et al., 2006) raising the possibility that these stimulus features account in part for the larger response of early visual cortex to shiny over matte, and the opposite pattern found in hMT+.

While this finding confirms the potential usefulness of these three motion cues in identifying reflectance properties, the current experimental design does not permit to identify the relative weight that these cues are given at this early stage of the visual analysis. This will be the topic of future experiments.

It is likely that simple cues like coherence or divergence play a less important role in motion-based material identification at intermediate levels of visual analysis compared to the early stages of visual processing. This idea is supported by the observed larger responses of LO1 to matte flows. However, this increased response might not necessarily indicate sensitivity to motion characteristics—such as coherence—*per se* (Larsson and Heeger, 2006), but rather reflect this area's sensitivity to 3D structure (Freeman et al., 2012). One hallmark of specular moving objects is that they frequently yield a non-rigid percepts. Thus, these stimuli may provide less information for computing 3D rigid shape from motion than diffusely reflecting moving objects—which might account for the observed pattern of responses.

Taken together, differences in motion coherence in shiny and matte flows appear to offer a fairly good account of the observed pattern of responses in areas V1 and hMT+, keeping in mind though that previous findings on coherence preferences of areas V1, V2, and hMT+ have been mixed. It is likely that it is a combination of factors such as direction variability, coherence and kinetic structure that is responsible for the observed pattern of cortical responses—either directly or potentially indirectly via altering other stimulus properties such as perceived surface brightness, contrast, or motion detection thresholds.

#### 4.2.2. Discrimination of matte vs. shiny flows across visual areas

Visual areas V1, V2, V3, V3A/B, and hMT+ were able to discriminate shiny and matte flows at levels significantly above chance, for both image and dot renders and within and between-class classification. What flow information is common to both photometric and kinematic flows that might explain the discriminative capabilities these visual areas? From the above discussion and stimulus measurements it appears that motion coherence, direction variability, and shape reliability are viable candidates, since these computations would all require to discount the intensity variations in photometric flow and thus be consistent with the extraction of a motion field. In line with this argument, we ruled out motion energy—a computation that does depend on photometric properties (Adelson and Bergen, 1985)—as an explanatory factor.

The response profile of these cortical areas thus suggests that the computation of surface material cannot simply discount structure, but that these two computations are interrelated. Intuitively this makes sense, given that 3D structure is needed, for example, to explain away (via boundary motion) a non-rigid interpretation of optic flow (see Supplementary Movies 6,7), or the interesting perceptual trade-off between perceived shininess and perceived rigidity of moving objects (Doerschner and Kersten, 2007; Zang et al., 2009, 2010; Doerschner et al., 2011a; Doerschner, 2013). Conversely, in some cases material inference may also influence perceived structure (Kersten et al., 1992).

### 4.3. Conclusion

Natural image flows carry information not only about structure, but also about material. Traditional studies of object motion have focused on structure-from-motion and have typically used very simple experimental manipulations such as varying coherence by randomizing the motions of subsets of dots. Dot flows largely miss the space of flows normally experienced and in particular which provide information for material. While were unable to identify regions of low and mid-level human visual cortex that respond preferentially to material structure from motion, we report the presence of several visual areas that modulate their activity with changes in specular reflectance flows.

Future work needs to be done to study brain mechanisms involved in the interaction between image flow properties and the conscious decisions about material. For example, Cant and Goodale (2007) have shown that attention to static object shape or material modulates different regions of the ventral stream, and more recently Wada et al. (2014) identified ventral and dorsal areas involved in both, the processing of image cues to glossiness as well as the perception of gloss in static images. More work is also needed to continue to quantify higher-order image regularities that capture characteristic motion patterns that support the dimensions along which humans can perceive object material qualities. For example, simple inspection of a rotating shiny object through an aperture illustrates the importance of boundary information and thus shape (Supplementary Movies 6, 7): without shape to “explain away” non-rigid interpretations, one often perceives non-rigid fluid flow.

### Conflict of interest statement

The authors declare that the research was conducted in the absence of any commercial or financial relationships that could be construed as a potential conflict of interest.

## Footnotes

^1^With the exception of area hMT+ which is known to saturate at lower contrasts.

^2^We equate variability with estimated variance of the motion direction histogram. Coherence is defined as similarity in motion direction *and* speed. The component that relates the two concepts is motion direction, however one can think of all possible constellations of high and low motion direction variability and high and low motion coherence. For example: A given dot motion field can have low motion direction variability and low coherence (if dots move at different speeds, but in the same direction) or low motion direction variability and high coherence (if dots move at the same speeds, in the same direction) or can have high motion direction variability and high coherence (half of the dots move at same speed in one direction and the other half at the same speed at the opposite direction, with non-overlapping dot trajectories) or can have high motion direction variability and low coherence (random direction dot motion).
