# Supplementary material for: Human visual cortical responses to specular and matte motion flows
Source: Front Hum Neurosci. 2015 Oct 21;9:579. doi: 10.3389/fnhum.2015.00579 (PMC4612507; doi:10.3389/fnhum.2015.00579)
Supplement: Supplementary file 10 [file Image1.PDF]

Supplementary Material: Human visual cortical responses to specular and matte motion flows

Tae-Eui Kam [1], **Damien J. Mannion** [2,3,4], **Seong-Whan Lee** [1,2,*], **Katja Doerschner** [5,6], and **Daniel J. Kersten** [2,4]

[1] *Department of Computer Science and Engineering, Korea University, Seoul, Republic of Korea*
[2] *Department of Brain and Cognitive Engineering, Korea University, Seoul, Republic of Korea*
[3] *School of Psychology, UNSW Australia, Sydney, NSW, Australia*
[4] *Department of Psychology, University of Minnesota, Minneapolis, MN, USA*
[5] *Department of Psychology, Bilkent University, Ankara, Turkey*
[6] *National Magnetic Resonance Research Center, Bilkent University ,Ankara, Turkey*

Correspondence*:
Seong-Whan Lee
Department of Brain and Cognitive Engineering, Korea University, 145, Anam-ro, Seongbuk-ku, Seoul, 02841, Republic of Korea, sw.lee@korea.ac.kr

SUPPLEMENTARY TABLES AND FIGURES

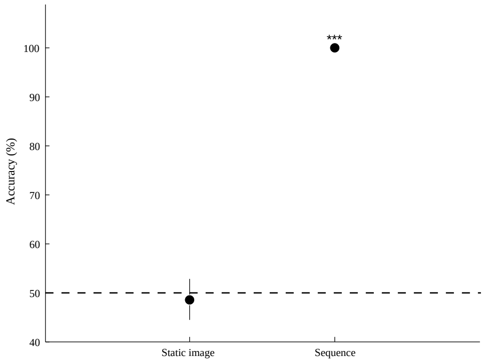

Supplementary Figure 1. Results of 2AFC (two-Alternative Forced Choice) judgement on static images and sequences of shiny/matte stimuli. We conducted a behavioral experiment to verify that observers were able to discriminate surface reflectance for the objects used in the fMRI experiment. We used the same photometric objects as described in Section 2.3 and presented in the same positions on the screen as in the fMRI experiment described in Section 2.4. On a given trial, shiny objects were presented on one side of the screen and matte objects on the other. The position of shiny and matte objects was randomized (left or right), and varied from trial to trial. In the motion case each object rotated back and forth twice, and in the static case, only the first frame of the motion sequence was presented for 2 seconds (Please see Supplementary Movies 8 and 9). After this, each participant was asked to indicate the side that they saw the shiny stimulus at. Seven participants judged each matte-shiny pair 4 times. Participants perfectly discriminated shiny and matte objects in the motion condition (Mean: 100, SE: 0, $p<0.001$ in an one sample t-test against a chance level of 50%) and were at chance level in the static condition (Mean=48.57, SE:4.19, $p>0.05$).

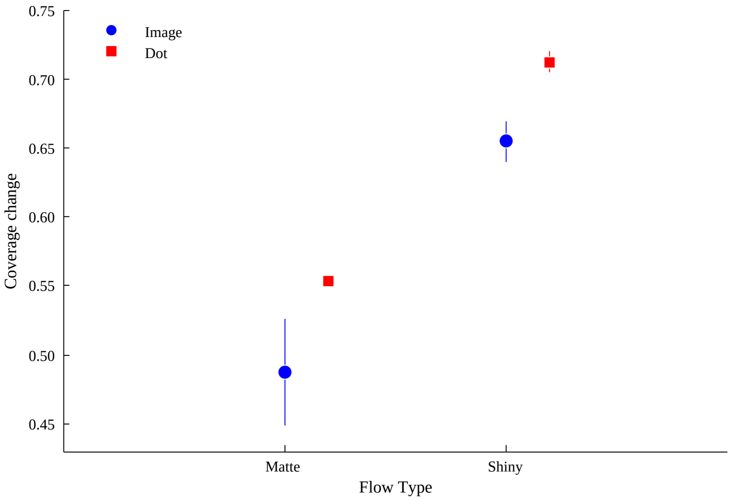

Supplementary Figure 2. Effect of flow type on coverage change for image and dot renders. Coverage quantifies the ratio of pixels with computed flow vectors to the number of all pixels. Coverage change is the reduction in coverage due to lengthening of the frame sequence (from 2 to 3 frames) quantifying the amount of trackability (see Doerschner et al., 2011a for a more detailed discussion of the motion cue).

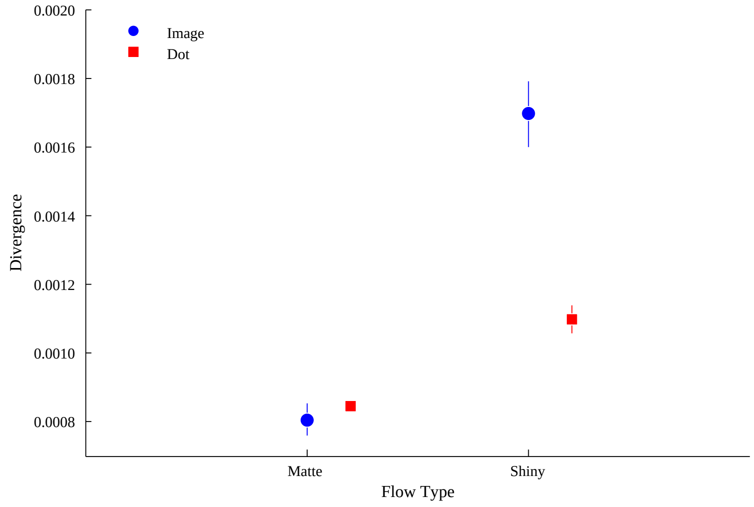

Supplementary Figure 3. Effect of flow type on divergence for image and dot renders. Divergence captures the strength of concavities and convexities that cause expansions and contractions in the flow field (see Doerschner et al., 2011a for a more detailed discussion of the motion cue).

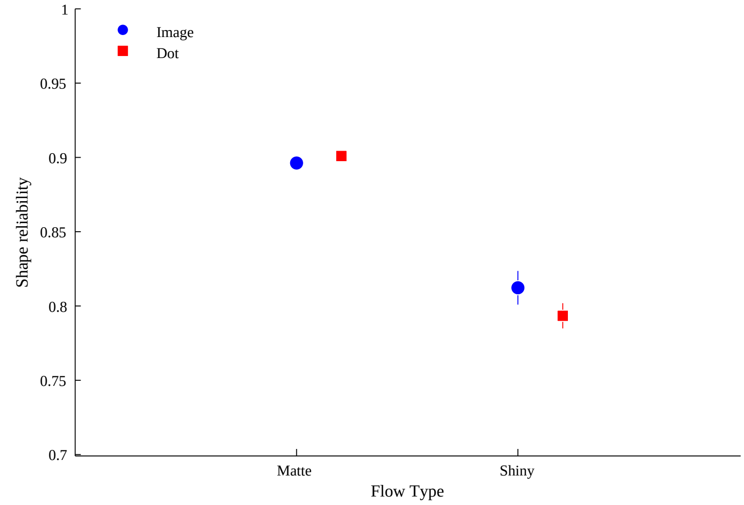

Supplementary Figure 4. Effect of flow type on shape reliability for image and dot renders. Shape reliability represents how consistently the optic flow vectors are constrained by epipolar geometry (see Doerschner et al., 2011a for a more detailed discussion of the motion cue).

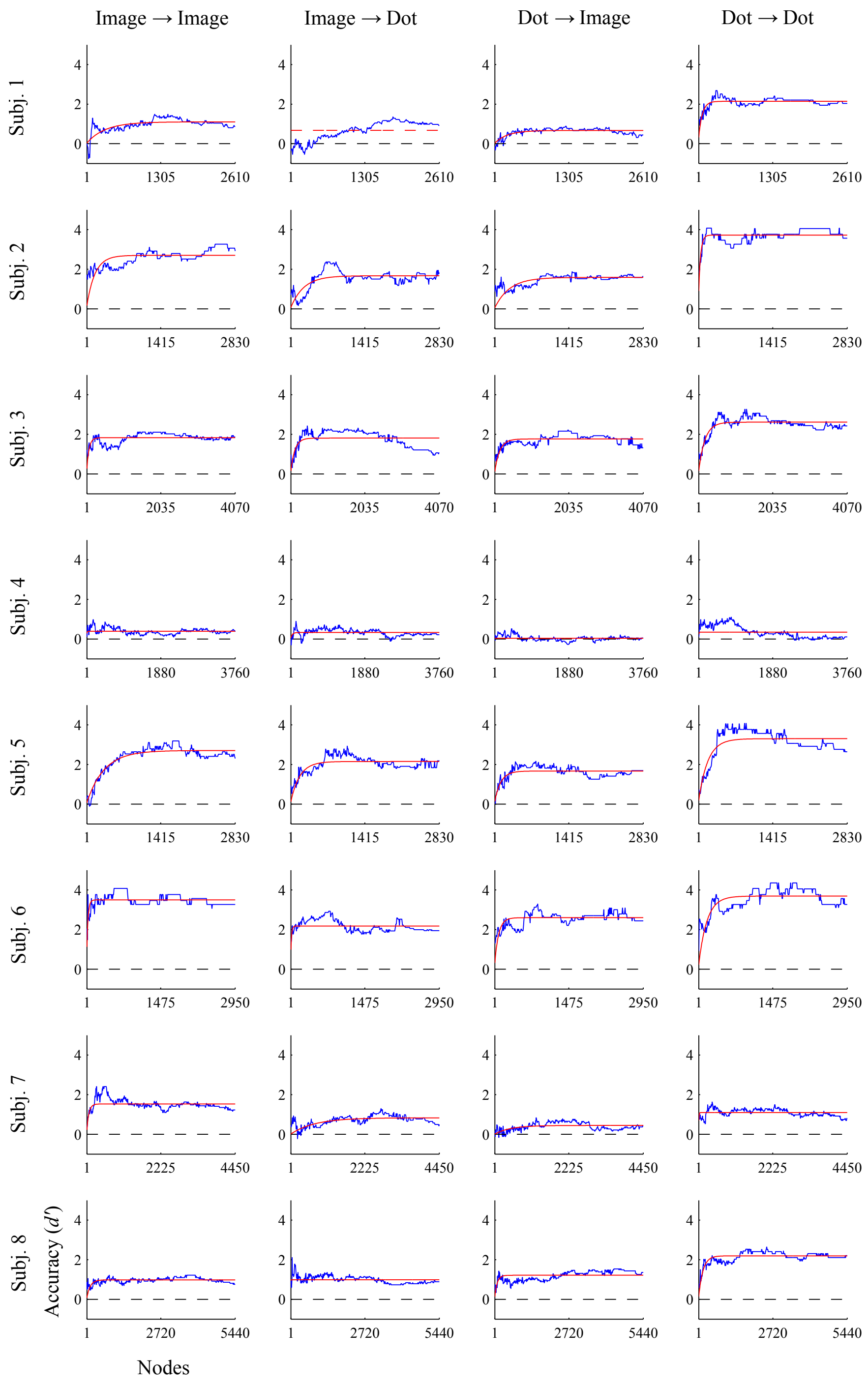

Supplementary Figure 5. Flow classification accuracy as a function of number of included features (nodes) for visual area V1. Each row shows a different participant, and each column shows a different training → test rendering class pair. Each blue line is the 12-fold average classification accuracy achieved, and the red line is a fitted exponential growth function. Dashed red lines indicate the mean accuracy across number of included nodes, in the case where such a function did not fit the accuracy data successfully.

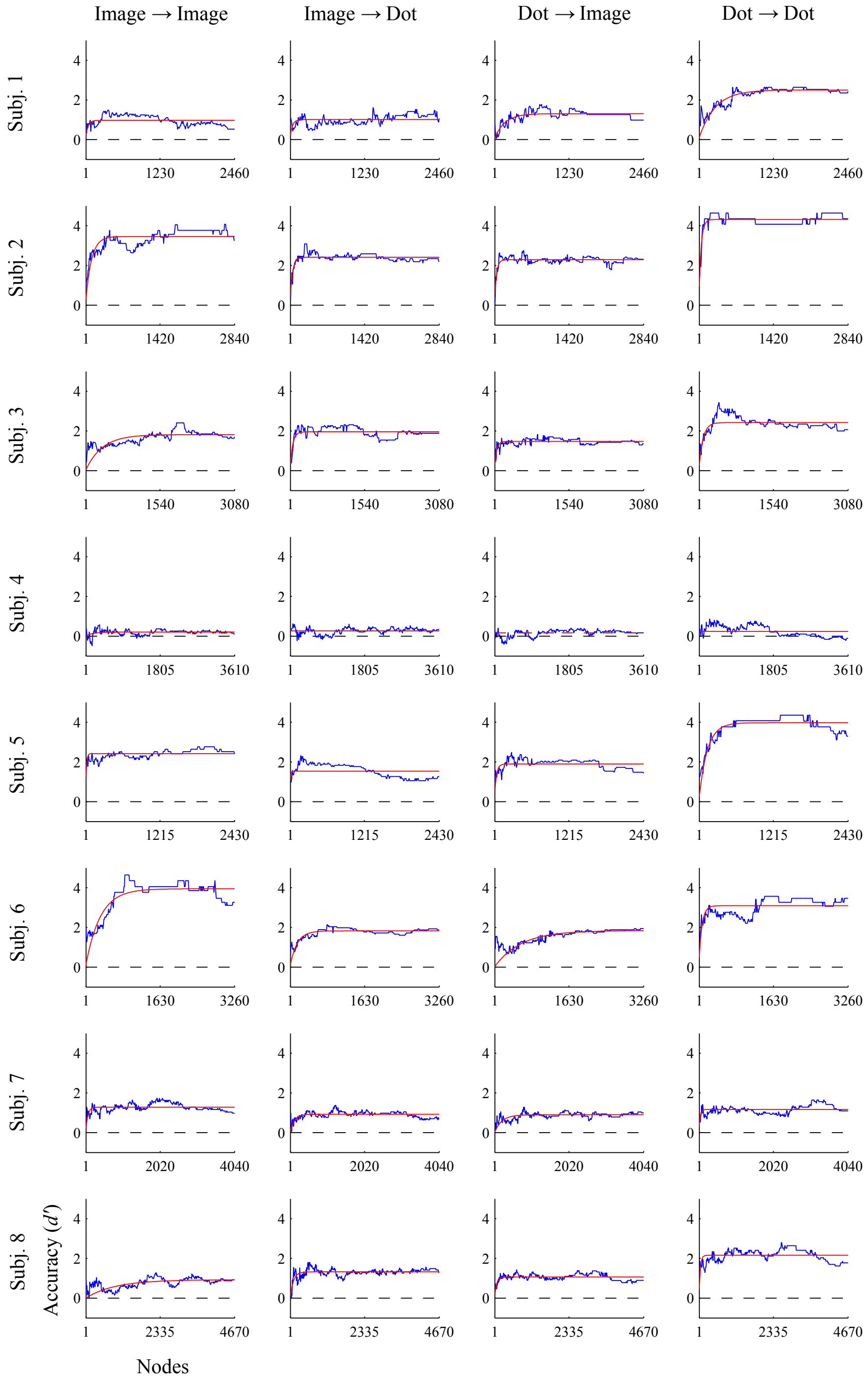

Supplementary Figure 6. Flow classification accuracy as a function of number of included features (nodes) for visual area V2. Each row shows a different participant, and each column shows a different training → test rendering class pair. Each blue line is the 12-fold average classification accuracy achieved, and the red line is a fitted exponential growth function. Dashed red lines indicate the mean accuracy across number of included nodes, in the case where such a function did not fit the accuracy data successfully.

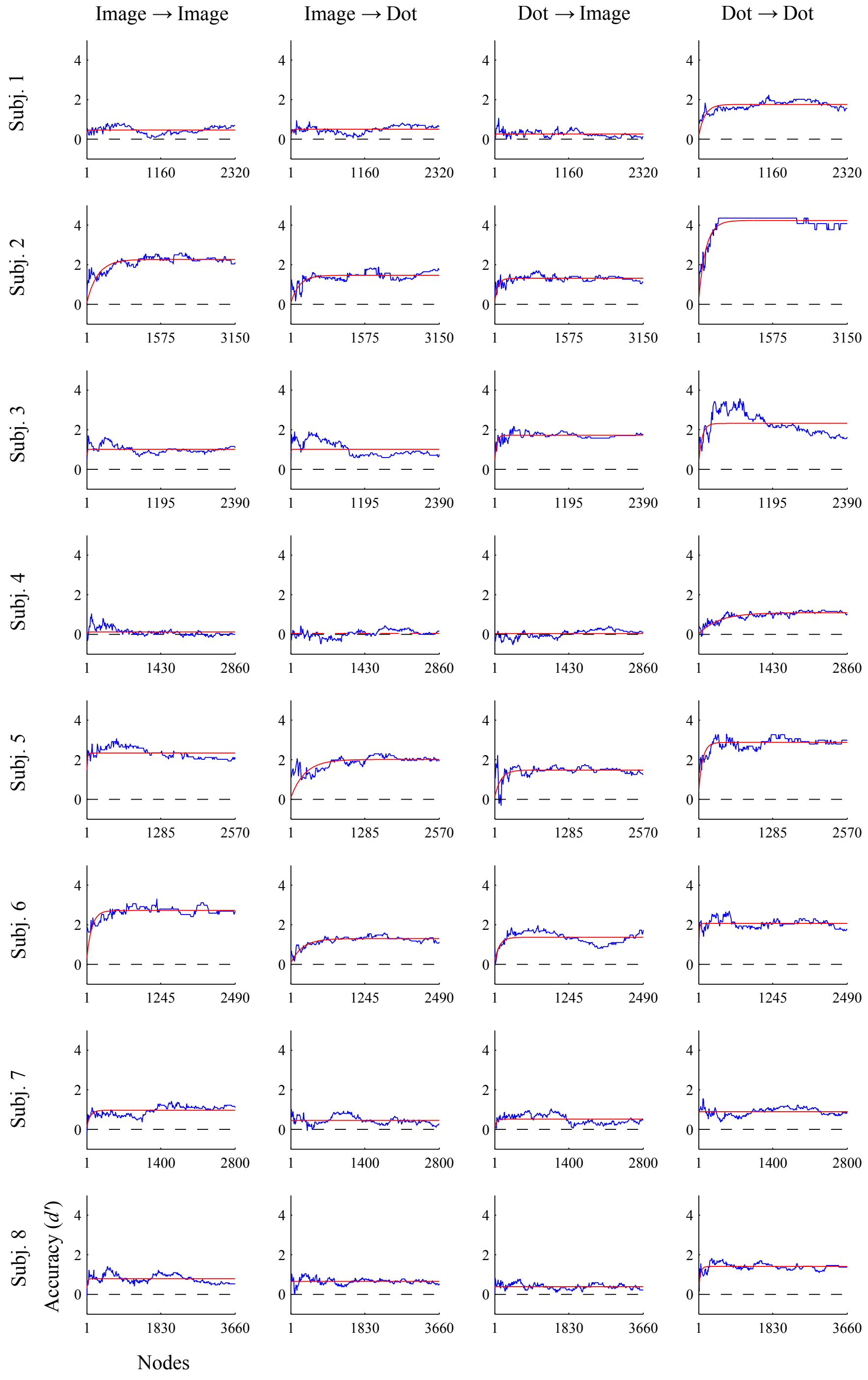

Supplementary Figure 7. Flow classification accuracy as a function of number of included features (nodes) for visual area V3. Each row shows a different participant, and each column shows a different training → test rendering class pair. Each blue line is the 12-fold average classification accuracy achieved, and the red line is a fitted exponential growth function. Dashed red lines indicate the mean accuracy across number of included nodes, in the case where such a function did not fit the accuracy data successfully.

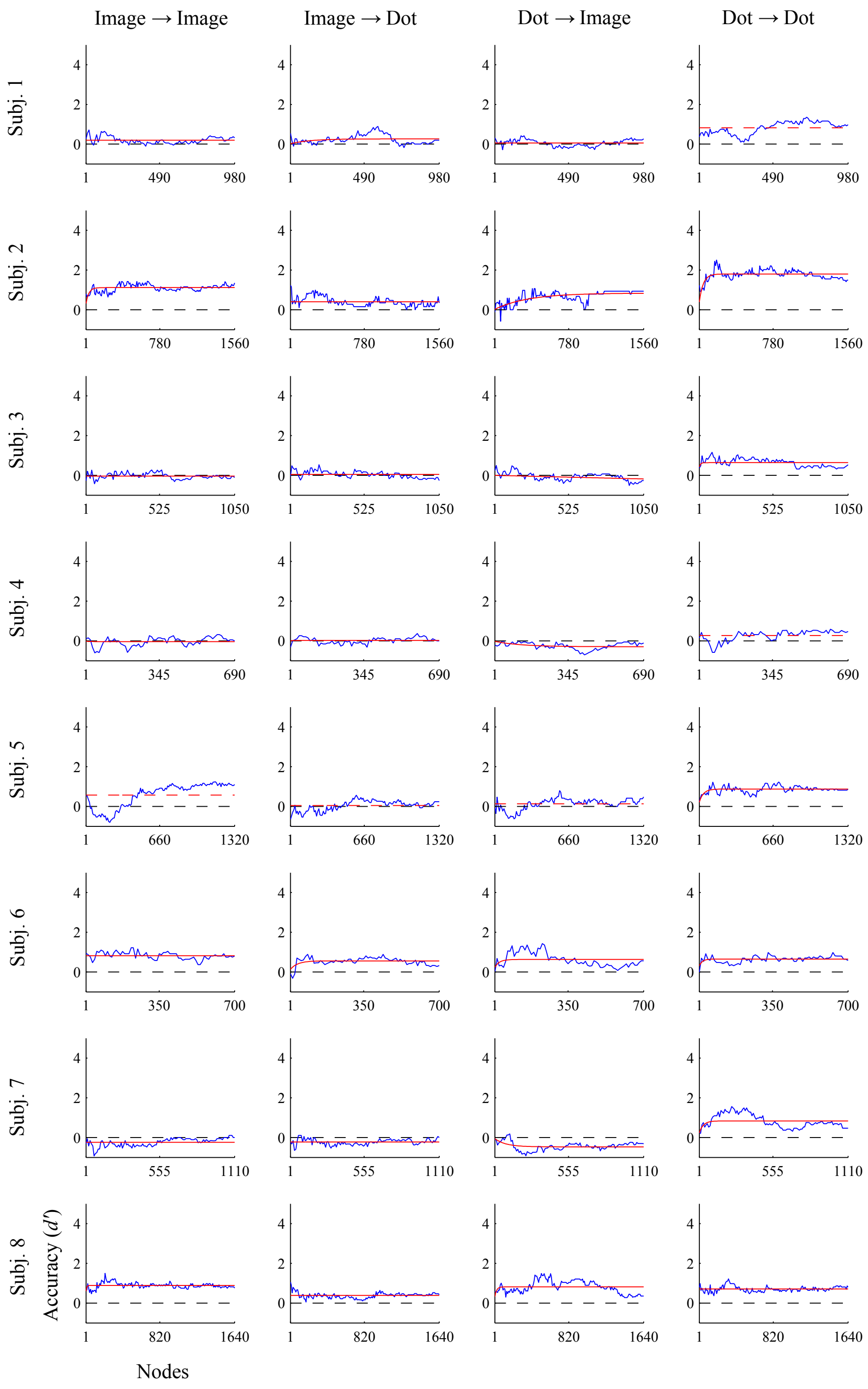

Supplementary Figure 8. Flow classification accuracy as a function of number of included features (nodes) for visual area hV4. Each row shows a different participant, and each column shows a different training → test rendering class pair. Each blue line is the 12-fold average classification accuracy achieved, and the red line is a fitted exponential growth function. Dashed red lines indicate the mean accuracy across number of included nodes, in the case where such a function did not fit the accuracy data successfully.

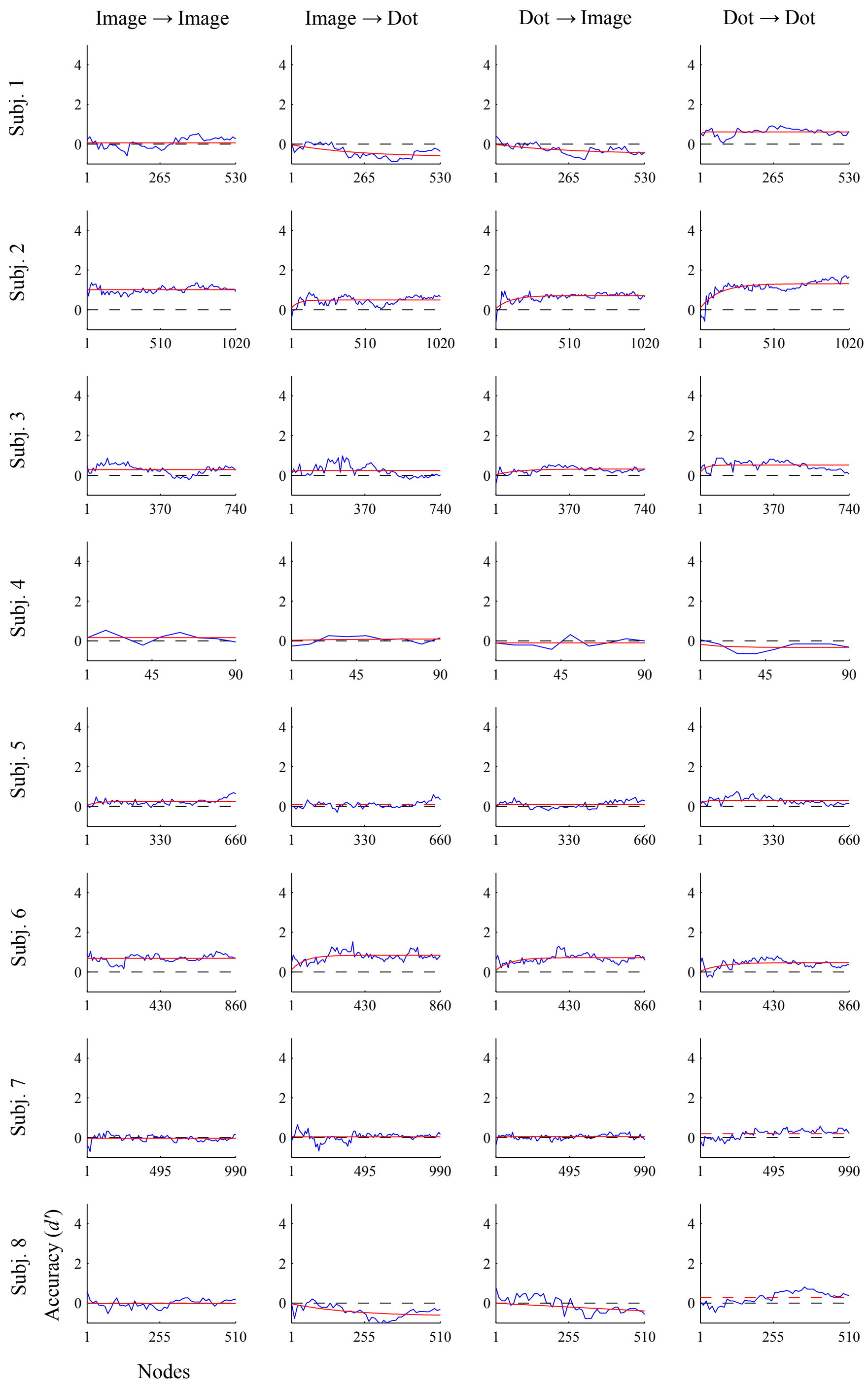

Supplementary Figure 9. Flow classification accuracy as a function of number of included features (nodes) for visual area VO1. Each row shows a different participant, and each column shows a different training → test rendering class pair. Each blue line is the 12-fold average classification accuracy achieved, and the red line is a fitted exponential growth function. Dashed red lines indicate the mean accuracy across number of included nodes, in the case where such a function did not fit the accuracy data successfully.

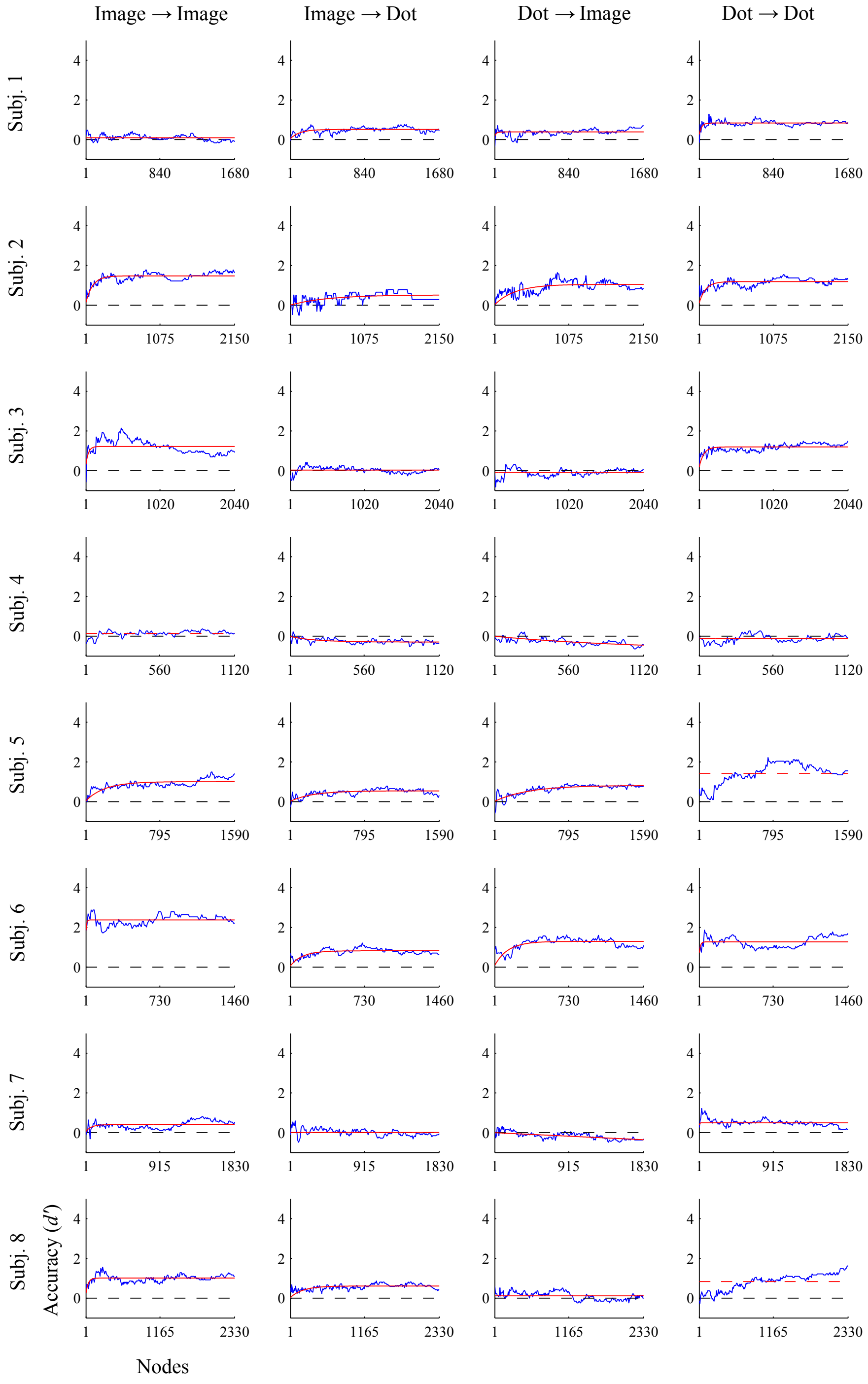

Supplementary Figure 10. Flow classification accuracy as a function of number of included features (nodes) for visual area V3A/B. Each row shows a different participant, and each column shows a different training → test rendering class pair. Each blue line is the 12-fold average classification accuracy achieved, and the red line is a fitted exponential growth function. Dashed red lines indicate the mean accuracy across number of included nodes, in the case where such a function did not fit the accuracy data successfully.

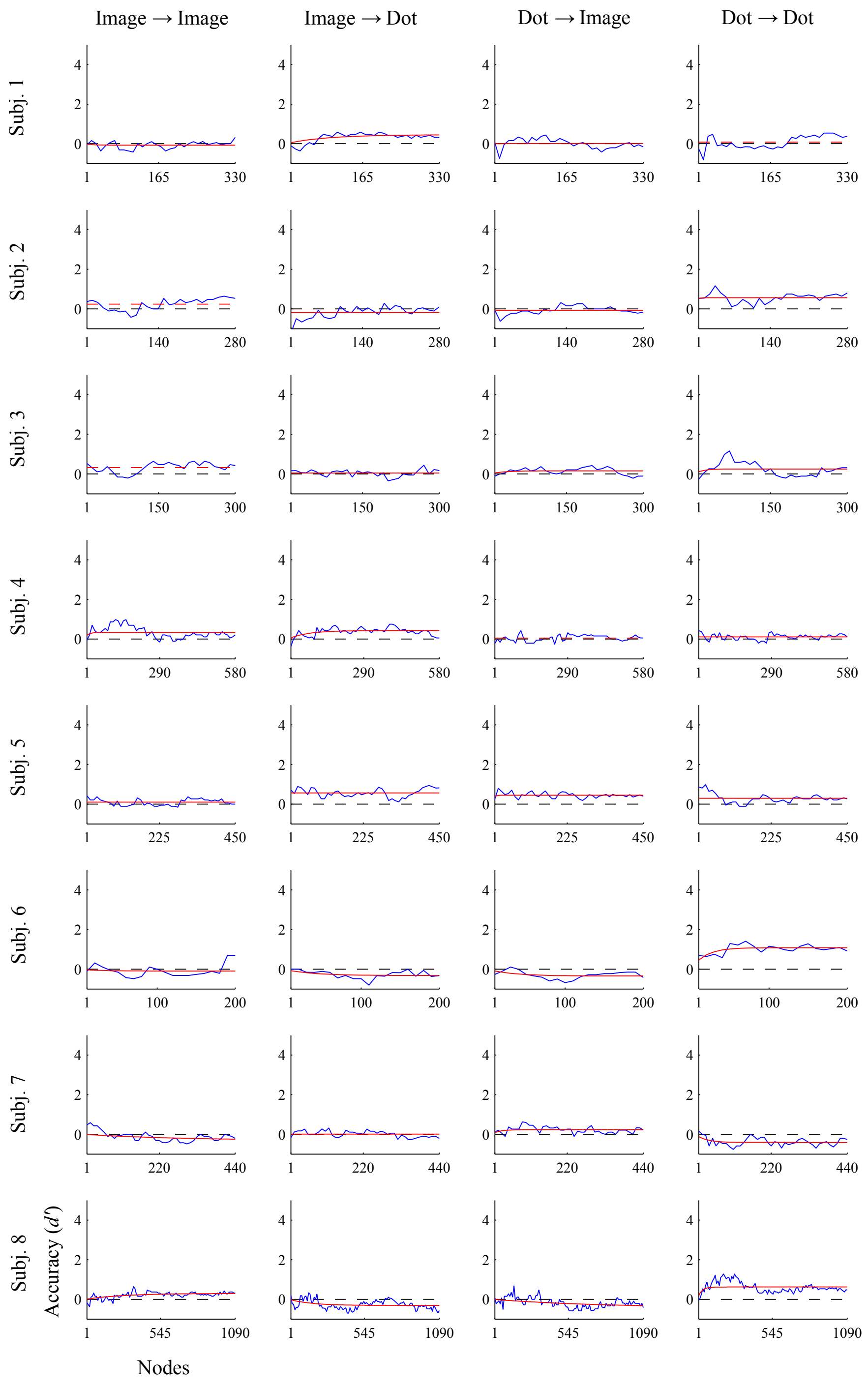

Supplementary Figure 11. Flow classification accuracy as a function of number of included features (nodes) for visual area LO1. Each row shows a different participant, and each column shows a different training → test rendering class pair. Each blue line is the 12-fold average classification accuracy achieved, and the red line is a fitted exponential growth function. Dashed red lines indicate the mean accuracy across number of included nodes, in the case where such a function did not fit the accuracy data successfully.

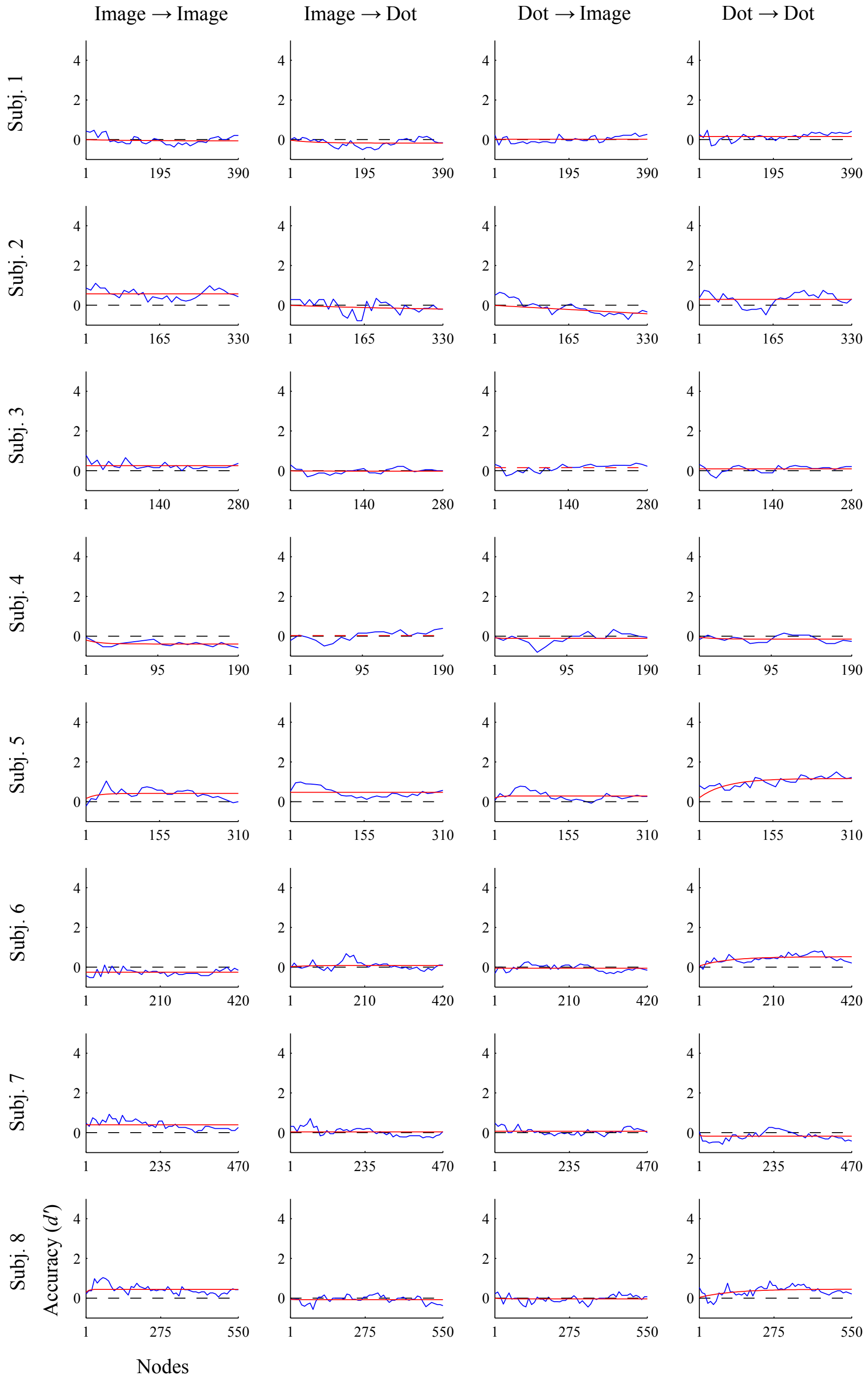

Supplementary Figure 12. Flow classification accuracy as a function of number of included features (nodes) for visual area LO2. Each row shows a different participant, and each column shows a different training → test rendering class pair. Each blue line is the 12-fold average classification accuracy achieved, and the red line is a fitted exponential growth function. Dashed red lines indicate the mean accuracy across number of included nodes, in the case where such a function did not fit the accuracy data successfully.

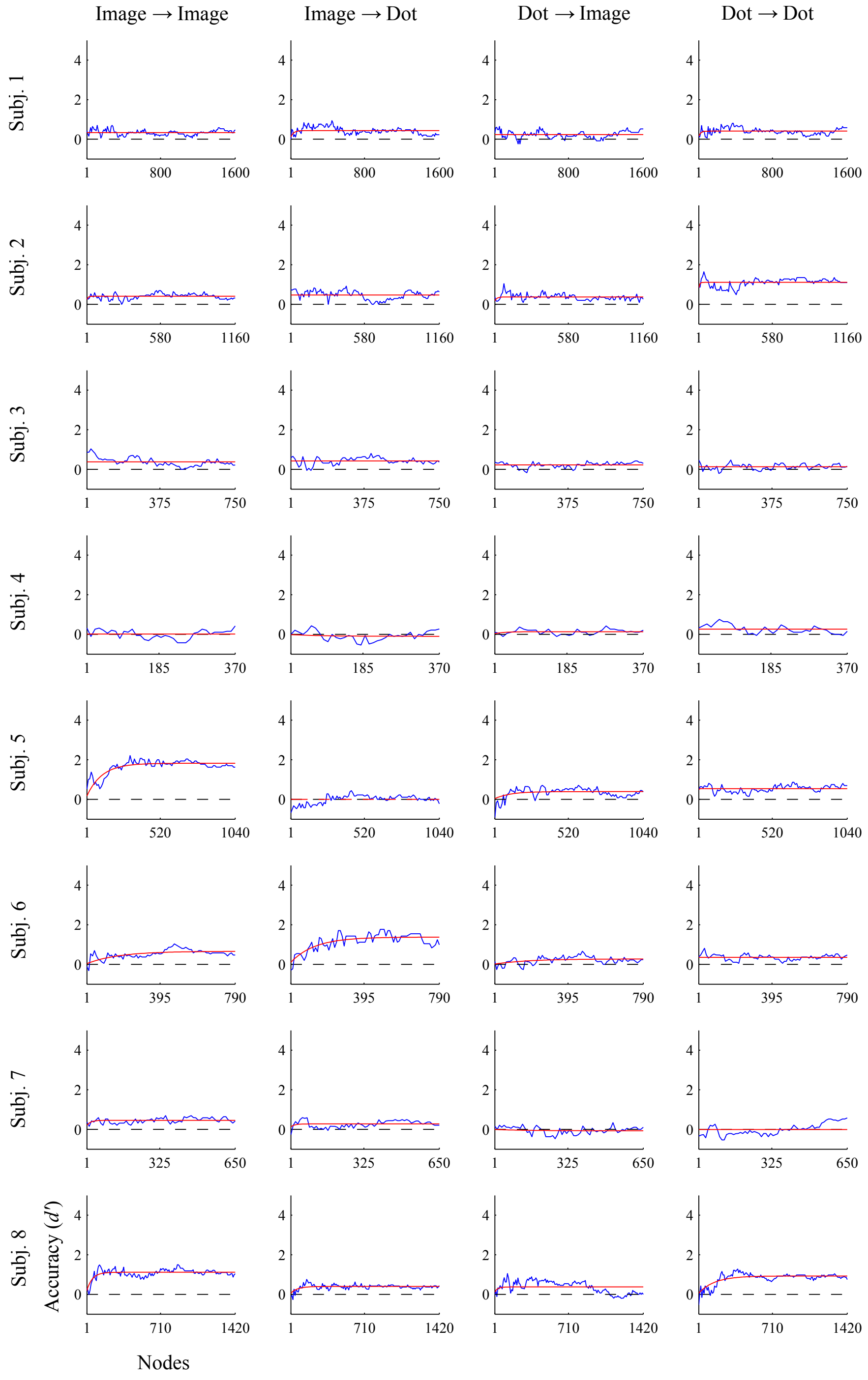

Supplementary Figure 13. Flow classification accuracy as a function of number of included features (nodes) for visual area hMT+. Each row shows a different participant, and each column shows a different training → test rendering class pair. Each blue line is the 12-fold average classification accuracy achieved, and the red line is a fitted exponential growth function. Dashed red lines indicate the mean accuracy across number of included nodes, in the case where such a function did not fit the accuracy data successfully.

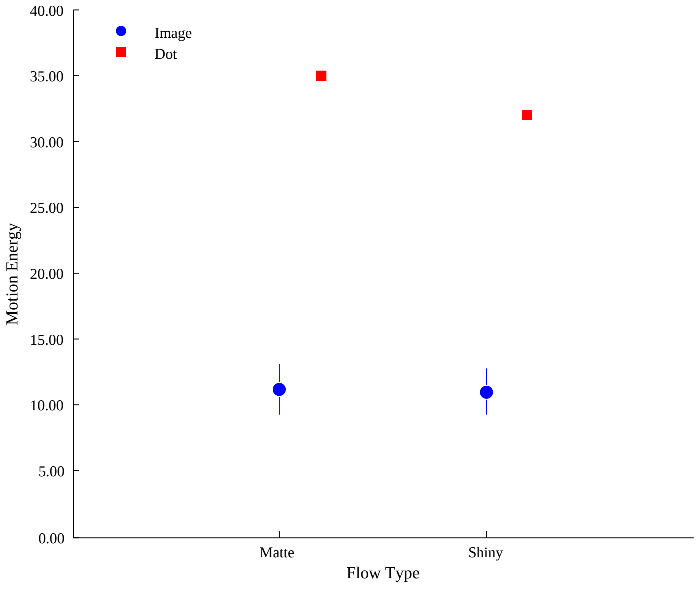

Supplementary Figure 14. Effect of flow type on motion energy for image and dot renders. Motion energy was calculated via the method described in “K.G. Derpanis and J.M. Gryn, *Three-Dimensional nth Derivative of Gaussian Separable Steerable Filters,* York University Technical Report CS-2004-05, November 22, 2004."

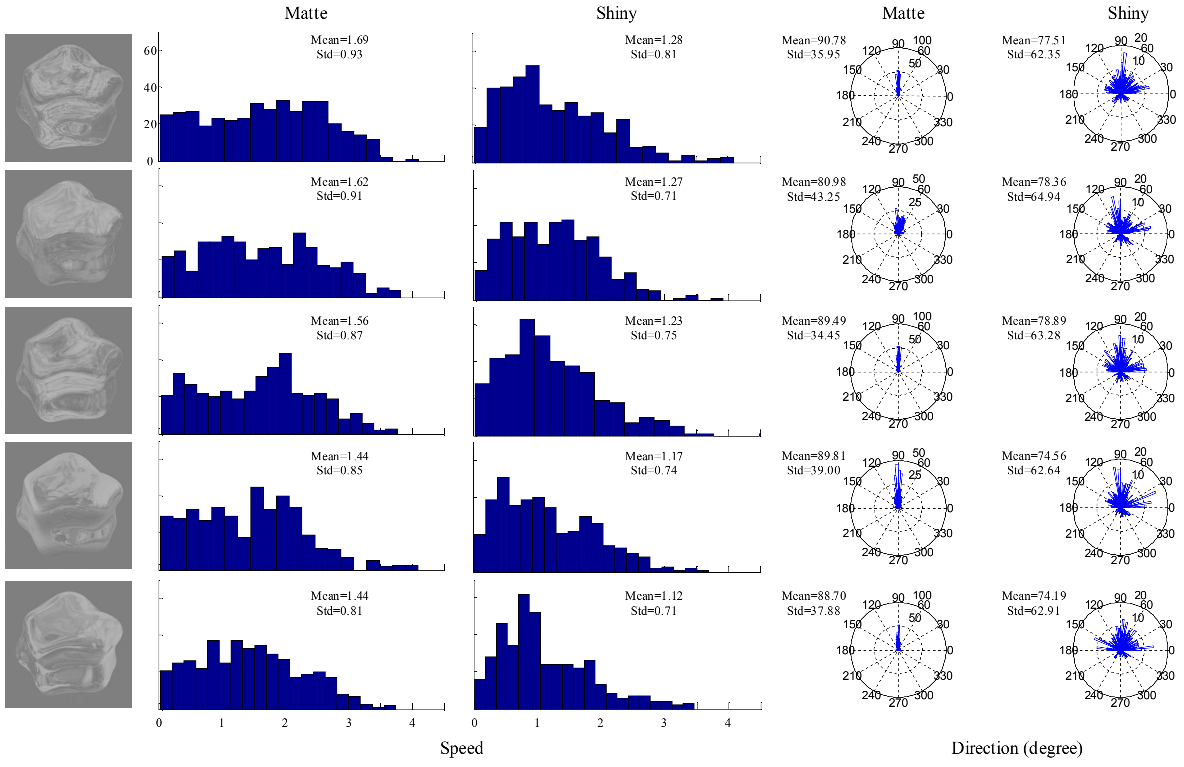

Supplementary Figure 15. Histograms of speed and direction between the first two frames for each object rotated around its horizontal axis.

Supplementary Table 1. Results of a two-samples t-test and one-way ANOVA on coverage change for each pair of stimulus classes. Coverage quantifies the ratio of pixels with computed flow vectors to the number of all pixels. Coverage change is the reduction in coverage due to lengthening of the frame sequence (from 2 to 3 frames) quantifying the amount of trackability (see Doerschner et al., 2011a for a more detailed discussion of the motion cue).

Compared	t-test			ANOVA		
stimulus classes	t_4	p	p_{FDR}	$F_{1,8}$	p	p_{FDR}
Matte Image vs. Shiny Image	6.639	0.003	0.005	16.063	0.004	0.006
Matte Image vs. Matte Dot	1.784	0.149	0.149	2.796	0.133	0.133
Matte Image vs. Shiny Dot	4.900	0.008	0.012	32.159	<0.001	0.001
Shiny Image vs. Matte Dot	7.461	0.002	0.005	43.620	<0.001	0.001
Shiny Image vs. Shiny Dot	2.653	0.057	0.068	12.195	0.008	0.010
Matte Dot vs. Shiny Dot	16.887	<0.001	<0.001	358.997	<0.001	<0.001

Supplementary Table 2. Results of a two-samples t-test and one-way ANOVA on divergence for each pair of stimulus classes. Divergence captures the strength of concavities and convexities that cause expansions and contractions in the flow field (see Doerschner et al., 2011a for a more detailed discussion of the motion cue).

Compared	t-test			ANOVA		
stimulus classes	t_4	p	p_{FDR}	$F_{1,8}$	p	p_{FDR}
Matte Image vs. Shiny Image	6.331	0.003	0.005	69.368	<0.001	<0.001
Matte Image vs. Matte Dot	0.664	0.543	0.543	0.632	0.450	0.450
Matte Image vs. Shiny Dot	3.444	0.026	0.031	22.531	0.001	0.002
Shiny Image vs. Matte Dot	9.904	0.001	0.002	77.317	<0.001	<0.001
Shiny Image vs. Shiny Dot	8.973	0.001	0.002	33.176	<0.001	0.001
Matte Dot vs. Shiny Dot	9.267	0.001	0.002	36.929	<0.001	0.001

Supplementary Table 3. Results of a two-samples t-test and one-way ANOVA on shape reliability for each pair of stimulus classes. Shape reliability represents how consistently the optic flow vectors are constrained by epipolar geometry (see Doerschner et al., 2011a for a more detailed discussion of the motion cue).

Compared	t-test			ANOVA		
stimulus classes	t_4	p	p_{FDR}	$F_{1,8}$	p	p_{FDR}
Matte Image vs. Shiny Image	6.487	0.003	0.004	69.368	<0.001	<0.001
Matte Image vs. Matte Dot	1.118	0.326	0.326	0.632	0.286	0.286
Matte Image vs. Shiny Dot	11.840	<0.001	0.001	22.531	<0.001	<0.001
Shiny Image vs. Matte Dot	9.903	0.001	0.001	77.317	<0.001	<0.001
Shiny Image vs. Shiny Dot	2.526	0.065	0.078	33.176	0.224	0.269
Matte Dot vs. Shiny Dot	21.207	<0.001	<0.001	36.929	<0.001	<0.001

Supplementary Table 4. The average (and SE) of motion energy (See Adelson and Bergen, 1985 and the website, `http://www.georgemather.com/Model.html`, for the details of the method), coverage change and divergence, and shape reliability. Coverage quantifies the ratio of pixels with computed flow vectors to the number of all pixels. Coverage change is the reduction in coverage due to lengthening of the frame sequence (from 2 to 3 frames) quantifying the amount of trackability. Divergence captures the strength of concavities and convexities that cause expansions and contractions in the flow field. Shape reliability represents how consistently the optic flow vectors are constrained by epipolar geometry (see Doerschner et al., 2011a for a more detailed discussion of these motion cues).

Class	Motion energy	Coverage change	Divergence	Shape reliability
Matte Image	11.21769 (1.92164)	0.48751 (0.03896)	0.00081 (0.00005)	0.89612 (0.00225)
Shiny Image	11.03156 (1.75521)	0.65455 (0.01482)	0.00169 (0.00010)	0.81214 (0.01141)
Matte Dot	34.97118 (0.17029)	0.55301 (0.00410)	0.00084 (0.00001)	0.90076 (0.00338)
Shiny Dot	31.98309 (0.12048)	0.71231 (0.00734)	0.00110 (0.00004)	0.79356 (0.00827)

Supplementary Table 5. Results of a two-samples t-test and one-way ANOVA on motion energy for each pair of stimulus classes.

Compared	t-test			ANOVA		
stimulus classes	t_4	p	p_{FDR}	$F_{1,8}$	p	p_{FDR}
Matte Image vs. Shiny Image	0.396	0.713	0.713	0.005	0.945	0.945
Matte Image vs. Matte Dot	13.238	<0.001	<0.001	151.606	<0.001	<0.001
Matte Image vs. Shiny Dot	11.011	<0.001	<0.001	116.315	<0.001	<0.001
Shiny Image vs. Matte Dot	14.768	<0.001	<0.001	184.291	<0.001	<0.001
Shiny Image vs. Shiny Dot	12.268	<0.001	<0.001	141.817	<0.001	<0.001
Matte Dot vs. Shiny Dot	26.431	<0.001	<0.001	205.178	<0.001	<0.001
